# New Information on *Tataouinea hannibalis* from the Early Cretaceous of Tunisia and Implications for the *Tempo* and Mode of Rebbachisaurid Sauropod Evolution

**DOI:** 10.1371/journal.pone.0123475

**Published:** 2015-04-29

**Authors:** Federico Fanti, Andrea Cau, Luigi Cantelli, Mohsen Hassine, Marco Auditore

**Affiliations:** 1 Dipartimento di Scienze Biologiche, Geologiche e Ambientali, Alma Mater Studiorum, Università di Bologna, Bologna, Italy; 2 Museo Geologico Giovanni Capellini, Alma Mater Studiorum, Università di Bologna, Bologna, Italy; 3 Office National Des Mines, Service Patrimoine Géologique, Tunis, Tunisia; 4 Museo Paleontologico Cittadino, Monfalcone, Gorizia, Italy; University of Pennsylvania, UNITED STATES

## Abstract

The rebbachisaurid sauropod *Tataouinea hannibalis* represents the first articulated dinosaur skeleton from Tunisia and one of the best preserved in northern Africa. The type specimen was collected from the lower Albian, fluvio-estuarine deposits of the Ain el Guettar Formation (southern Tunisia). We present detailed analyses on the sedimentology and facies distribution at the main quarry and a revision of the vertebrate fauna associated with the skeleton. Data provide information on a complex ecosystem dominated by crocodilian and other brackish water taxa. Taphonomic interpretations indicate a multi-event, pre-burial history with a combination of rapid segregation in high sediment supply conditions and partial subaerial exposure of the carcass. After the collection in 2011 of the articulated sacrum and proximalmost caudal vertebrae, all showing a complex pattern of pneumatization, newly discovered material of the type specimen allows a detailed osteological description of *Tataouinea*. The sacrum, the complete and articulated caudal vertebrae 1–17, both ilia and ischia display asymmetrical pneumatization, with the left side of vertebrae and the left ischium showing a more extensive invasion by pneumatic features than their right counterparts. A pneumatic hiatus is present in caudal centra 7 to 13, whereas caudal centra 14–16 are pneumatised by shallow fossae. Bayesian inference analyses integrating morphological, stratigraphic and paleogeographic data support a flagellicaudatan-rebbachisaurid divergence at about 163 Ma and a South American ancestral range for rebbachisaurids. Results presented here suggest an exclusively South American Limaysaurinae and a more widely distributed Rebbachisaurinae lineage, the latter including the South American taxon *Katepensaurus* and a clade including African and European taxa, with *Tataouinea* as sister taxon of *Rebbachisaurus*. This scenario would indicate that South America was not affected by the end-Jurassic extinction of diplodocoids, and was most likely the centre of the rapid radiation of rebbachisaurids to Africa and Europe between 135 and 130 Ma.

## Introduction

The partial skeleton of a rebbachisaurid sauropod was discovered in the fall of 2011 by Mr. A. Bacchetta during a geological investigation at the Jebel El Mra locality (Tataouine Governorate, Tunisia) led by the University of Bologna. Prospecting activities at the site followed previous discovery of fossil-rich beds and scattered crocodilian and fish remains littering the Aptian-Albian deposits exposed in the area. A first excavation carried out in collaboration with the Office National des Mines (ONM) resulted in the acquisition of the sacrum and the first five caudal vertebrae, that were consequently transported to the Musée de l’Office National des Mines in Tunis. Unfortunately, these elements were severely vandalized after their transportation to the capital city: the unstable political situation of the recent years resulted in terrible damages at the cultural heritage of this country, including paleontological specimens. Only in the spring of 2012, thanks to the direct involvement of the Tunisian authorities, it was possible to access the specimen and start the difficult preparation of damaged elements. Despite a big investment of time and resources, more than 200 fragments pertaining to the sacrum and proximal caudal vertebrae were not returned to the original conditions. Nevertheless, the restored material allowed to formally instituting *Tataouinea hannibalis* as a new genus and species of rebbachisaurid sauropod [1]. The new sauropod is characterized by an extensive pattern of postcranial pneumatisation in most of the recovered skeleton. In particular, *Tataouinea* shows, for the first time among non-avian dinosaurs, an ischial pneumatic foramen, further corroborating the presence of a bird-like system of air sacs in sauropods [1]. Additional information based on field notes and pictures, measurements, and quarry maps taken during the first excavation are presented in this study in order to carefully reconstruct some of the vandalized skeletal elements. A new field expedition in the spring of 2013 led to the collection of the fully articulated rest of the tail (caudal vertebrae 6–17) as well as further sedimentological and paleontological investigations at the El Mra locality.

## Geological Setting

**Fig 1:**
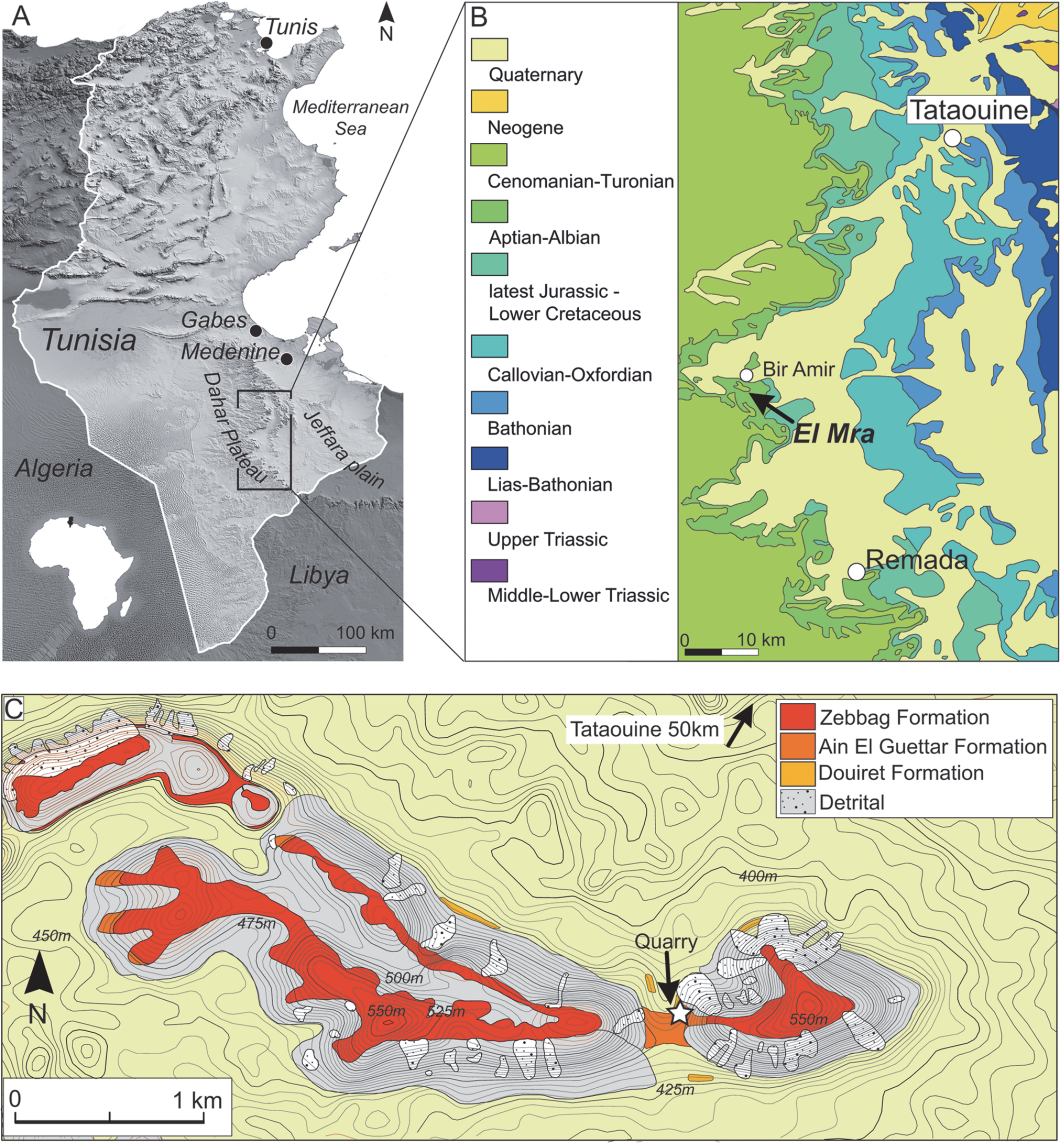
The Tataouine basin in southern Tunisia. A, reference map of the Tataouine region in southern Tunisia; B, simplified geological map of the study area showing the distribution of Mesozoic deposits and the El Mra locality near the village of Bir Amir. C, detailed topographic and geological map of the El Mra locality.

Since the first geological and paleontological reports published more than a century ago by Léon Pervinquière and other French geologists and paleontologists [2–6], the sedimentary beds of the Dahar escarpment in southern Tunisia have been known as a source of pivotal information on the Early Cretaceous ecosystems of northern Africa. The results of geological, paleontological and biogeographic investigations that followed tens of scientific expeditions in the area are largely presented and discussed in the literature ([7–16] and references therein). Although the exposed Late Jurassic-Early Cretaceous alternation of shallow-marine, littoral, and non-marine deposits named “*Continental Intercalaire*” by Kilian in 1931 [3] is nowadays documented over much of northern Africa [17–20], the southern Tunisian outcrops provide unequalled stratigraphic and paleontological data (Fig 1). Several major canyons and gorges as well as numerous minor drainage systems that cut the Dahar Plateau to the pediment that slopes toward the east forming the western margin of the Jeffara plain characterize the study area, located in the Tataouine Governorate. Therefore, the overall geomorphology is characterized by *mesa*-like structures that locally expose up to 150 meters of Jurassic and Cretaceous deposits, historically considered to represent sequential periods of time and different environments. The “*Continental Intercalaire*” exposures in the Tataouine region are represented, in ascending order, by the Oxfordian-lower Aptian Merbah el Asfer Group (Bir Miteur, Boulouha, and Douiret formations) and the overlying lower Albian Ain El Guettar (Chenini, Oum ed Diab and Rhadouane members) and Cenomanian-Turonian Zebbag formations (Kerker and Gattar members) (we refer to [14] for a detailed revision of stratigraphic units and chronostratigraphic framework).

### The Jebel El Mra section

**Fig 2:**
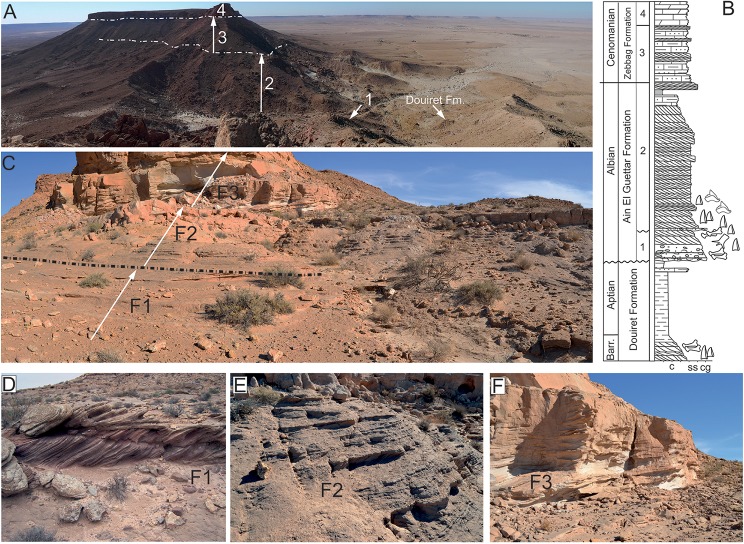
Stratigraphy at the El Mra locality. A, panoramic view of the El Mra mesa-like morphology. 1. Chenini Mbr., 2. Oum Ed Diab Mbr., 3. Keker Mbr., 4. Gattar Mbr. B, simplified field-log of the El Mra section showing the stratigraphic occurrence of vertebrate and plant remains. C, facies distribution in the Chenini-Oum Ed Diab transition. F1, high-energy, fluvial sandy bar deposits; F2, low-angle shoreface deposits; F3, fine-grained tidal deposits with flaser-like structures. D, E, F, field photographs of the different deposits exposed at the El Mra locality. *T*. *hannibalis* was recovered from the F1 beds.

The Jebel El Mra site is located approximately 50 km to the south-west of Tataouine, and 5 km to the south of the Bir Amir village (Fig 1). The carbonate deposits of the Zebbag Formation cap this west-east oriented *mesa*, and substantial erosion has exposed underlying deposits for 120 meters to the uppermost, clay-dominated beds of the Douiret Formation (Fig 2). The articulated elements of *Tataouinea hannibalis* were collected in the basal deposits of the Oum ed Diab Member, approximately 1.5 meters above the fossil-rich conglomerate that marks the erosive contact between the Chenini and the overlying Oum ed Diab members [1, 14, 16] (Fig 2B). This basal marker bed, that crops out with a patchy pattern in the entire Tataouine basin, yielded a rich and diverse vertebrate fauna that include elasmobranchs, actinopterygii, coelacanthiformes, crocodyliforms, rare pterosaurs and dinosaurs. However, as this faunal assemblage occurs within transgressive lag deposits on transgressive erosive surface, part of the preserved taxa may pertain to the underlying Chenini Member. Lacking any direct evidence to detail the age of these beds, [14] assigned an early Albian age to the lower Oum ed Diab Member deposits based on basin-scale paleontological, stratigraphic, and palynological evidence. The skeleton of *T*. *hannibalis* was preserved in a two meter thick, unconsolidated sandy interval characterized by sizeable, almost unidirectional (to the south-west) cross-bedding structures (Fig 2C). This interval is capped by meter-thick, carbonate-cemented, horizontally-bedded sandstones: horizontal planes are characterized by extensive hematitic crusts and common, decimetre-scale rhizocretions, both interpreted as pedogenetic features. A significant reduction in flow energy is observed in the overlying deposits that consist in horizontally-bedded, fine- to coarse-grained, largely unconsolidated sandstones. High-angle, cross bedding structures observed in the most basal beds are gradually replaced by fining up sequences of tabular and fine-grained sandstones alternating ripple marks, herringbones cross-bedding and sporadic symmetric, wave-formed ripples.

#### Vertebrate remains

The basal conglomeratic beds of the Oum ed Diab Member sampled at the El Mra locality as well as other localities in the Tataouine basin yielded a rich and diverse vertebrate fauna that include marine elasmobranchs (*Tribodus tunisiensis*, *Lissodus* sp., *Diabodus tataouinensis*, *Retodus semiplicatus*, *Leptostyrax macrorhiza*, *Scaphanorinchus* sp., and *Onchopristis dunklei*), bony fish taxa (*Lepidotes* sp., *Mawsonia* sp.,*Ceratodus* sp. and *Neoceratodus* sp.), crocodyliforms (*Sarcosuchus* sp., cf. *Araripesuchus* sp., and cf. *Hamadasuchus* sp.), and dinosaurs (carcharodontosaurids, spinosaurids, abelisaurids, titanosauriforms, and ornithopods) [10, 14–16, 21, 22]. At the El Mra locality, the vast majority of identifiable elements pertains to crocodyliforms (a few notosuchian-like and abundant neosuchian remains), whereas non-sauropod dinosaurs are represented by spinosaurids theropods that may represent two distinct clades, i.e., Baryonychinae and Spinosaurinae [16]). The skeletal material of *T*. *hannibalis* represents the sole articulated dinosaur specimen ever collected from the Oum ed Diab Member and generally from Tunisia.

#### Facies analysis and paleoecology

The sandy deposits of the Oum ed Diab Member at the El Mra locality are interpreted as fluvial sandy bars within a vast, estuarine system characterized by high sediment supply an accommodation rate, as well as relatively high-energy hydraulic regime (Fig 2C–2F). The occurrence of *in situ* plant roots (possibly indicating a patchy, mangrove-like vegetation) are consistent with sub-aerial to low water depth conditions. Furthermore, molds, tubules, and rhizocretions, as well as extensive hematitic hard grounds support the development of thin paleosoils typical of arid to xeric environment [23–25]. These lower sandy units gradually shift to shoreface beds interbedded with tidal flat/foreshore deposits, thus interpreted as a vast embayment characterized by tidal influence and dominated by marine taxa (i.e. elasmobranchs).

## Material and Methods

All elements collected from the Jebel El Mra locality are housed in the Musée de l’Office National des Mines, Minestère de L’Industrie et de la Technologie, 24 Rue 8601, La Charguia, 2035 Tunis, Tunisia, under the accession numbers ONM DT 1–48. Field data are housed at the Museo Geologico Giovanni Capellini (MGGC), via Zamboni 63, 40126 Bologna, Italy. All permits concerning the material described in this study are regulated by the ‘Accorde Cadre 1764—prot. 381’ signed on Dec 23rd 2010 between le l’Office National Des Mines (Dr. Abdelbaki Mansouri) and the Dipartimento di Scienze Biologiche, Geologiche e Ambientali, Alma Mater Studiorum, Università di Bologna (Prof. Giovanni Gabbianelli) which fulfilled with all relevant regulations.

In 2012 and 2013, high-resolution digital models of all sacral neural arches, caudal vertebrae, ilia and ischia, as well as of the entire 2013 quarry were acquired in Tunis by combining laser scanner (Next Engine ScanStudio HD Pro, alignment of the scans) and hi-resolution photogrammetry, and consequently elaborated using Agisoft PhotoScan Professional, and Meshlab. These data were finally combined in order to obtain photogrammetric virtual reconstruction of the main quarry and scaled replicas (1:3) of the entire caudal series and ischia using a Makerbot 3D printer at the Dipartimento di Scienze Biologiche, Geologiche e Ambientali of the Università di Bologna, Italy. These data were combined with field pictures and measurements and comparison with the sacral region of other sauropods (e.g., *Diplodocus carnegii*, MGGC 8723) in order to reconstruct the damaged section of the sacrum. Field data and replicas of skeletal remains are housed at the Museo Geologico Giovanni Capellini, Bologna. At the time of writing, all elements collected from the Jebel El Mra locality are housed in the Musée de l’Office National des Mines in Tunis under the accession numbers ONM DT 1–48.

Sacral vertebrae nomenclature follows [26]; caudal vertebrae laminae and fossae nomenclatures follow [27] and [28]; pneumatic features terminology follows [29, 30].

Phylogenetic taxonomy follows [31] when not emended (see below).

## Taphonomy of *Tataouinea hannibalis*

**Fig 3:**
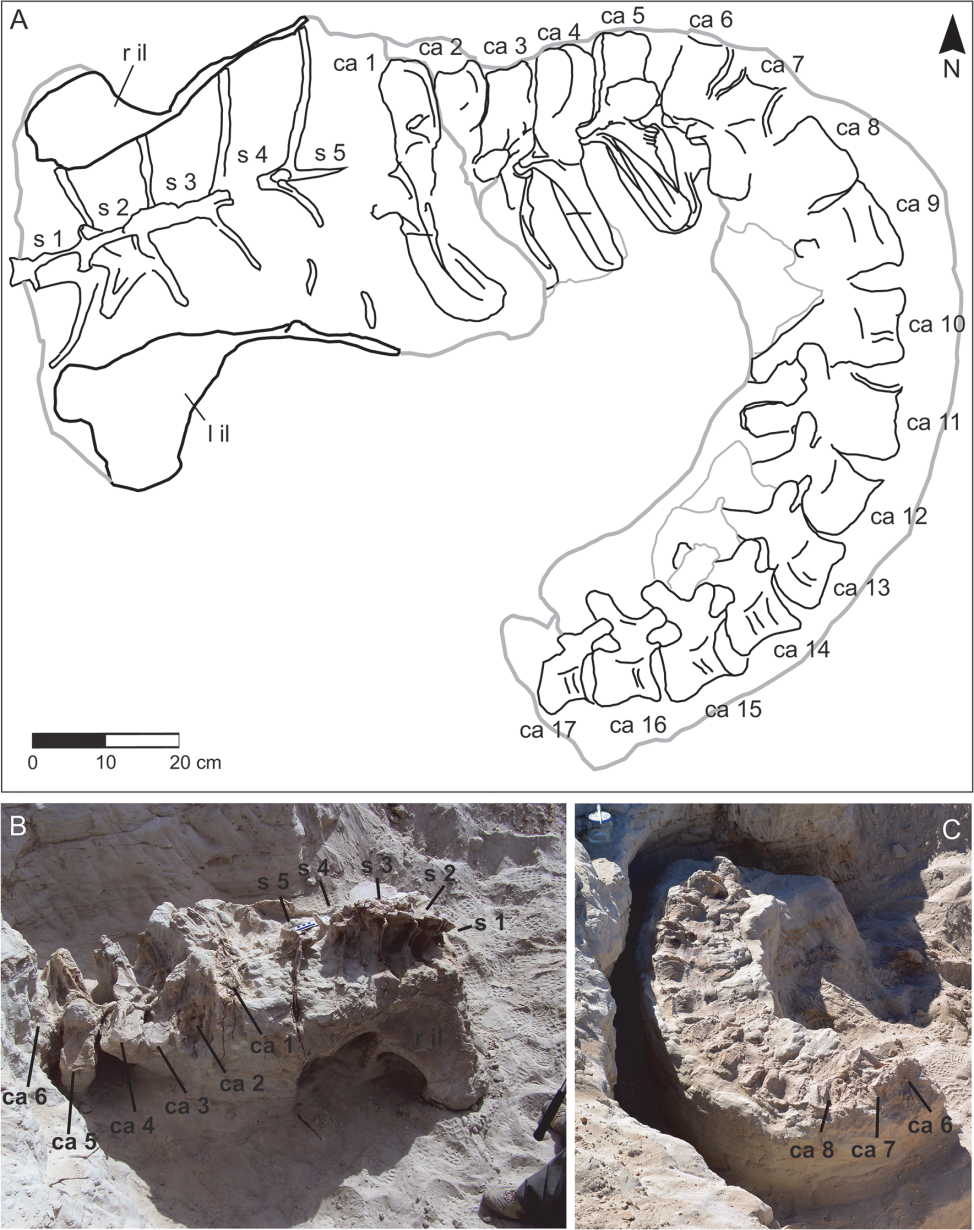
Preserved elements of *Tataouinea hannibalis* (ONM DT 1–48). A, quarry map showing the orientation of collected elements. B, field photograph of the elements collected at the end of 2011, and C, of elements collected in 2013. Ca, caudal vertebra 1–17; S, sacral vertebra 1–5; r il, right ilium; l il, left ilium.

The type specimen of *T*. *hannibalis* represents the first articulated Mesozoic archosaur from Tunisia and one of the best-preserved dinosaur specimens ever collected in northern Africa (Fig 3A). Unfortunately, a water channel that possibly eroded away the majority of the skeleton delimits the excavated area. However, based on available data it is not possible to determine if the missing parts of the skeleton were: 1. eroded away in recent times, 2. not preserved at the time of the original burial, or 3. buried as disarticulated elements over a wider area. Although preserved elements show no evidence of major post-mortem transportation, flow direction measurements taken on cross-bedding structures at the site suggest that sediment deposited onto the skeleton may have come toward the animal from a posterior to right side direction. The absence of the chevrons of the caudal series that conflicts with the fully articulated and nicely preserved vertebrae and sacral elements also support this interpretation. The sacrum was preserved lying on its ventral surface, a condition that strongly suggests a rapid segregation of this element from hydraulic flows and high sediment supply. In craniodorsal view, the fused neural spines of the sacral vertebrae are vertically oriented with respect to the paleo-ground (Fig 3B). The overall preservation of skeletal remains was excellent, although sacral neural spines were partially eroded in their dorsal tip. As a possible consequence of relatively short-termed subaerial exposure, the right side of the caudal vertebrae is more deteriorated than the left counterpart. Although the sacral centra were obscured in any view in the field, their preservation indicates they were fully articulated with the neural arches and spines. Similarly, the displacement of both ischia was trivial, although only the proximal end of these elements was preserved. Considering the overall preservation of the skeleton, the absence of pubis and legs is puzzling as investigation at the site in the sediments surrounding and underlying the sacrum and the tail did not indicate any further skeletal element. The tail curves cranially to the left of the body, lying on the left side, with an angle of almost 90 degrees with respect to the sacrum (Fig 3C): the complete series of the first seventeen caudal vertebrae was collected at the excavation site. With the exception of the first caudal vertebra that was unexpectedly found detached from the caudal series and laying above the caudal part of the sacrum, all caudal vertebrae were fully articulated. No clear evidence of recent erosive event was noticed at the end of the caudal series, thus the lack of distal caudal vertebrae is interpreted as a displacement of these elements prior to the complete burial. Detailed sedimentological observation at the excavation site supports that skeletal elements were not fully buried in a single event. In particular, the most ventral part of the sacrum was buried by a single, high-energy event (indicated by the high-angle, cross-bedding stratification) that possibly left the dorsal section of the body, at the level of the sacral neural spines, exposed. A second event, represented by the cemented, tabular sand beds exposed in the upper section of the excavation site, finally buried remaining elements. A single, invertebrate feeding trace that did not affect the bony material was found in association with the skeleton. As the effects of invertebrate colonies are well documented in the literature [32–38] a single trace is consistent with a rapid segregation of *T*. *hannibalis* from the external environment. Lacking evidence of scavenging and deterioration of skeletal elements, the skeleton was most likely fully buried over a restricted period of time.

## Systematic Palaeontology

Dinosauria

Saurischia

Sauropoda

Rebbachisauridae [39]

Khebbashia clade nov.

### Phylogenetic definition

The least inclusive clade including *Limaysaurus tessonei*, *Nigersaurus taqueti* and *Rebbachisaurus garasbae*.

### Remarks

According to Article 36 of the International Code of Zoological Nomenclature, *Rebbachisaurus* is the eponymous genus of the ranked clade Rebbachisaurinae [39]. Furthermore, *Limaysaurus* and *Nigersaurus* are the eponymous genera of, respectively, Limaysaurinae [31], and Nigersaurinae [31]. Regardless to the relative relationships among these genera and the inclusiveness of the ranked clades anchored to them, we suggest the introduction of the unranked clade name Khebbashia for the least inclusive clade containing all these taxa and excluding more basal forms (see [1, 31, 40]). Note that Khebbashia cannot be a junior synonym of Rebbachisauridae under any alternative phylogenetic hypothesis, as the latter is a branch-based clade (i.e., the most inclusive clade containing *Rebbachisaurus garasbae* but excluding *Diplodocus longus* [31]) whereas the former is a node-based clade.

### Etymology

From “Khebbash” or “Khebbache”, a Moroccan seminomadic tribe that inhabited the region where the first rebbachisaurid specimen was found [40].

Rebbachisaurinae [39] (Nigersaurinae *sensu* [31])

### Diagnosis

Middle and caudal dorsal neural arches with caudal centroparapophyseal lamina; proximal caudal vertebrae with a ventral interprezygapophyseal lamina; proximal caudal vertebrae with a lamina bisecting the prezygapophyseal centrodiapophyseal fossa; proximal caudal vertebrae with triangular lateral processes on the neural spine [41–43].

### Phylogenetic definition

The most inclusive clade including *Rebbachisaurus garasbae* and excluding *Limaysaurus tessonei*.

### Remarks

Both phylogenetic analyses by [40] and our study (see below), have incorporated information of *Rebbachisaurus* absent in previous phylogenies, and consistently recover the latter closer to *Nigersaurus* than *Limaysaurus*. Therefore, following the taxonomy of [31], *Rebbachisaurus* is a member of the subfamily-ranked clade Nigersaurinae. According to Articles 36 and 63.1 of the International Code of Zoological Nomenclature, the subfamily-ranked clade including *Rebbachisaurus* has to be Rebbachisaurinae [40]. Therefore, we consider Nigersaurinae [31], as a junior synonym of Rebbachisaurinae [39].

Tataouinea hannibalis [1]

### Holotype

ONM DT 1–48, sacrum, caudal vertebrae 1 to 17, both ilia, both ischia.

### Type locality and horizon

Ain el Guettar Formation, Oum ed Diab Member, Jebel El Mra, Tataouine Governorate, southern Tunisia; early Albian. Estuarine to shallow marine deposits showing fining-upward sequences of fine-graded sandstones with herringbone cross-bedding, symmetrical wave-formed ripples and discontinuous clay lenses.

### Diagnosis (emended)

Rebbachisaurine sauropod dinosaur with unique combination of: completely fused sacral neural spines 1–3; poorly laminated cranial sacral neural spines, extensively laminated and semicamellate caudal sacral neural spines; elliptical foramen in lateral surface of fourth sacral neural spine penetrating the camerate sector of the spine; proximalmost five caudal vertebrae with elliptical pleurocoel placed at mid-height in the lateral surface of centrum that leads to a camerate internal pneumatisation; proximal caudal prezygapophyses not joined ventrally by a horizontal lamina; pneumatic foramen in the spinoprezygapophyseal fossa of proximal caudal vertebrae; pneumatic foramen in the prezygospinodiapophyseal fossa of proximal caudal vertebrae; “lateral lamina” in proximal caudal neural spines is “inverted Y”-shaped, formed by the spinoprezygapophyseal and spinodiapophyseal laminae, eventually merging dorsally with the spinopostzygapophyseal lamina and bordering a triangular fossa; caudal vertebrae 13–15 with shallow elliptical fossae on lateral surface of centrum; pubic peduncle of ilium hollowed by a large chamber; ischium with large elliptical foramen in the medial surface of the iliac peduncle (autapomorphy).

## Description

### Sacrum

**Fig 4:**
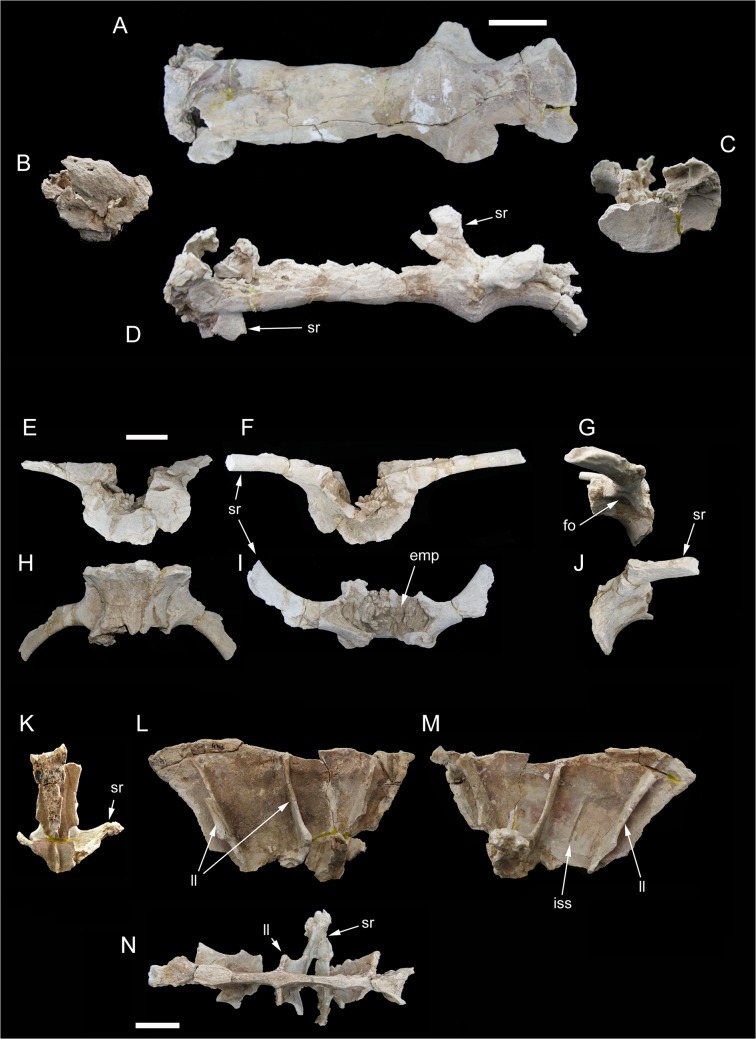
Partial sacrum of *Tataouinea hannibalis*. Partial sacral centra 1–4 in ventral (A), cranial (B), caudal (C) and lef lateral (D) views. Partial sacral centrum 5 in caudal (E), cranial (F), right lateral (G), ventral (H), dorsal (I) and left lateral (J) views. Scale bars: 10 cm. Abbreviations: emp, extramural pneumatisation; fo, fossa; iss, interspinal suture scar; ll, lateral lamina; sr, sacral ribs.

**Fig 5:**
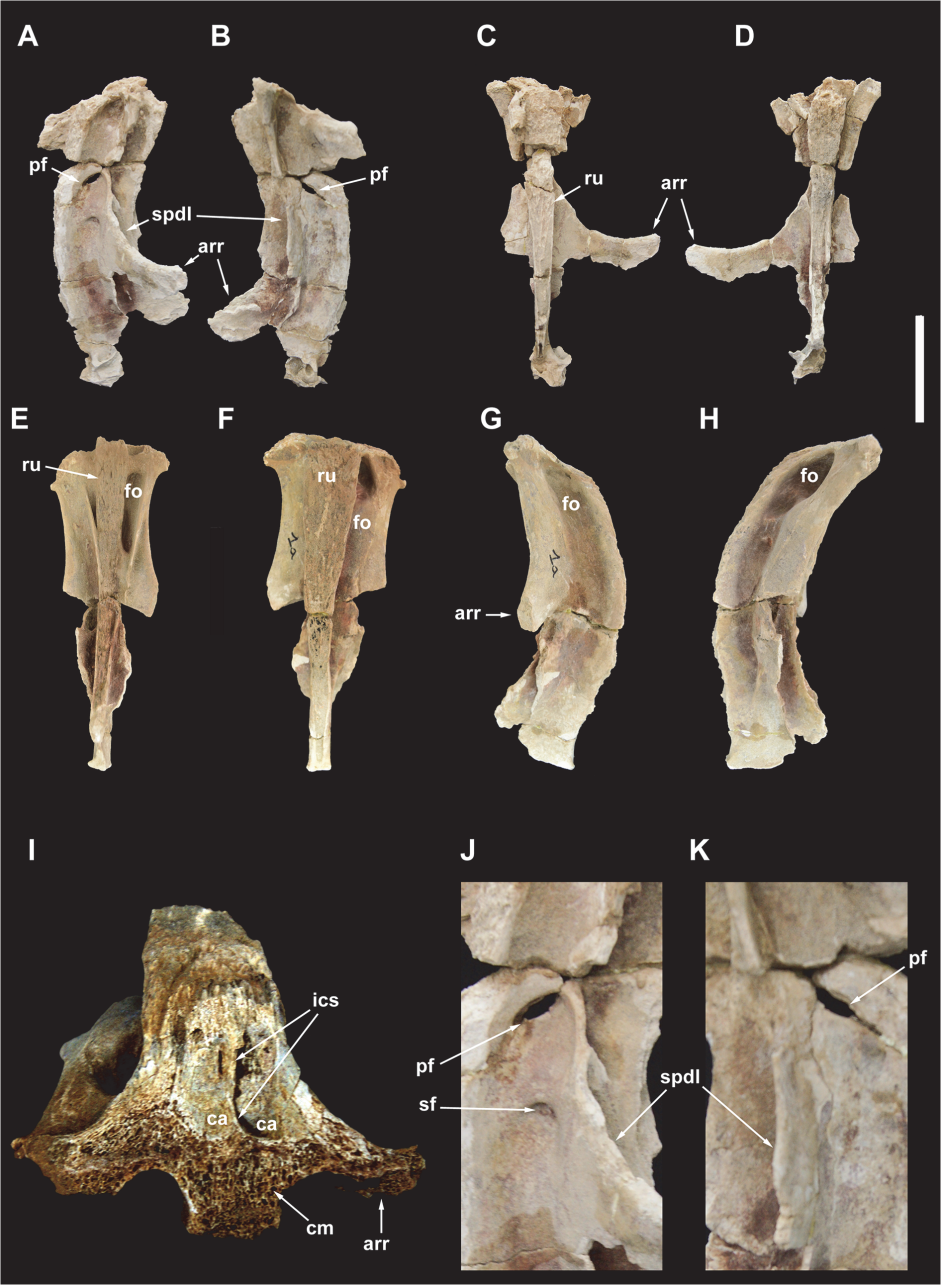
Sacral neural spines 4 and 5 of *Tataouinea hannibalis*. Sacral neural spine 4 in left lateral (A), right lateral (B), cranial (C) and caudal (D) views. Sacral neural spine 5 in caudal (E), cranial (F), right lateral (G) and left lateral (H) views. Cross section of sacral neural spine 4 (I). Details of sacral neural spine 4 pneumatisation in left lateral (J) and right lateral (K) views. Scale bar: 10 cm. Abbreviation: arr, alar ramus of rib; ca, camera; cm, camellae; fo, fossa; ics, intercamerate septum; pf, pneumatic foramen; ru, rugosities; sf, semilunate fossa; spdl, spinodiapophyseal lamina.

**Fig 6:**
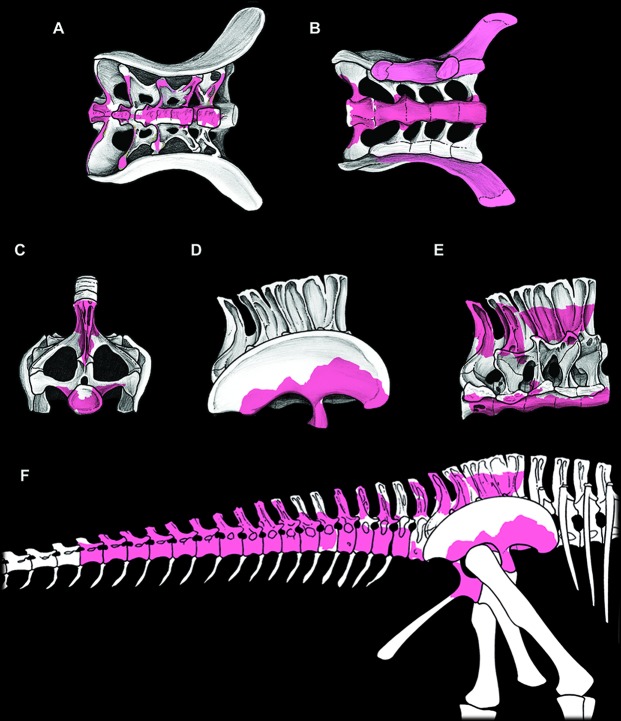
Sacrum with associated ilia, and reconstruction of known elements of *Tataouinea hannibalis*. Sacrum in dorsal view (A), ventral view (B), caudal view (C), right lateral view with ilium associated (D) and with ilium removed (E). In (B), the peduncles of the right ilium are removed. Skeletal reconstruction of caudosacral region of *Tataouinea hannibalis* (F). Recovered elements in colour. A full skeletal reconstruction is shown in S1 File.

The sacrum (Figs 4–6) is the part of the preserved skeleton that has suffered the most important damage. The preserved elements include the ventral half of the fused sacral centra 1 to 4 (Fig 4A–4D), part of the ventral half of the isolated fifth sacral centrum (Fig 4E–4J), the ventral half of the completely fused sacral neural spines 1 to 3 (Fig 4K–4N), and fragments of sacral neural spines 4 and 5 (Fig 5). Intercentral junctions show a progressive degree of fusion, caudo-cranially directed: intercentral junction s4/5 is open, s3/4 is tightly fused with clearly discernible suture, whereas both s2/3 and s1/2 are completely obliterated with no clear sutures visible. The s1-4 centra form a roughly straight series in both lateral and dorsoventral views. Intercentral junction s3/4 is transversely and dorsoventrally larger than the other junctions. The minimum transversal diameter of all centra is comparable along the series. Consequently, sacral centrum 4 appears as hourglass-shaped in ventral view, sacral centrum 3 appears as a truncated cone, whereas the other centra are more roughly cylindrical. The proximal part of both acetabular rami of the sacral rib 4 are preserved. The costovertebral junction 4 is completely obliterated. The acetabular rami are dorsoventrally expanded and join cranially the intercentral junction s3/4. A large chamber occupies most of the internal space of the fused centra, due to extensive extramural pneumatisation [29, 30]. The isolated sacral centrum 5 is partially preserved. The caudal intercentral facet and the dorsal half of the centrum is missing. The cranial intercentral facet is mostly eroded away. The centrum is a truncated cone, with the cranial end wider and deeper than the preserved posterior margin. The lateral surface of the centrum shows shallow elliptical fossae ventral to the costovertebral junctions. Internally, the centrum is extensively pneumatised by a large chamber. The proximal parts of the acetabular rami of sacral ribs are fused to the centrum. The preserved portions of the sacral ribs are projected caudally.

Only a limited part of the sacral neural arches is preserved. The largest element includes the ventral half of co-ossified neural spines 1–3, and the proximodorsal parts of both alar rami of sacral ribs and spinodiapophyseal laminae. In dorsal view, the element describes a cruciate pattern due to the intersection of the joined “neural spines + prespinal laminae” complex (directed axially) and the “ribs + spinodiapophyseal laminae” complex (directed transversely). The neural spines are moderately narrow and laminar. In lateral view, poorly developed ridges, oriented dorsoventrally, mark the interspinal joining between consecutive neural spines. The alar rami of the sacral ribs are directed dorsolaterally, forming an angle of about 60° with the dorsoventral axis of the neural spines. The lateral surfaces of the neural spines are relatively flat, lacking lateral fossae or pneumatic features. The spinodiapophyseal laminae are prominent, and describe a concave curve in cranial/caudal view. The dorsomedial margins of the preserved spinodiapophyseal laminae expand laterally, suggesting that, when complete, the neural spine was dorsally expanded. The ventral part of sacral neural spine 4 is preserved. It is similar to neural spines 1 to 3 in overall shape and preservation, and differs in showing a more complex pattern of fossae and recesses in the lateral surfaces. Cross section of the neural spine shows a semicamellate internal pneumatisation (Fig 5I). Both pre- and postspinal laminae are present. The prespinal lamina is thick and gently flares dorsally in cranial view. The cranial surface of the prespinal lamina is scarred by a discontinuous rugose pattern of ossified interspinous ligaments, oriented dorsoventrally. The postspinal lamina is comparable in overall features to the prespinal lamina. The spinodiapophyseal laminae run along mid-length of the lateral surfaces bounding two elliptical fossae (here termed “cranial fossa” and “caudal fossa”). The cranial fossa is excavated on both sides by an accessory fossa, placed at mid-height of the preserved spine (Fig 5J). This fossa is deeper in its dorsal end, and perforated on both sides by an elliptical foramen with its long axis oriented cranioventrally-caudodorsally. On the left side of the spine, a shallower crescentic fossa is placed caudoventrally to the accessory fossa described above, at the level of the alar ramus of the rib base. No equivalent fossa is present on the right side of the spine (Fig 5K). The caudal fossae on the lateral surfaces of the neural spine lack accessory pneumatisation. The semicamellate internal pneumatisation of the neural spine includes a cranial camerate sector (formed by a pair of large chambers placed symmetrically) that communicates with the external surface through the elliptical foramina described above, and a caudal camellate sector. Part of the ventral half of the fifth sacral neural spine is preserved. It is similar to the fourth sacral neural spine, in showing prominent pre- and postspinal laminae, and in the presence of the spinodiapophyseal laminae, the latter running dorsoventrally and bordering two elliptical fossae on the lateral surfaces. The pneumatic excavations on the lateral surfaces of the fifth sacral neural spine is less developed than in the previous neural spine, and no accessory fossae or foramina are present (Fig 5G and 5H). The most notable feature of the fifth sacral neural spine is the development of a pair of elliptical fossae on both the cranial and caudal surfaces (Fig 5E and 5F). These fossae are dorsoventrally oriented, bounded medially by the pre/postspinal laminae and laterally by a couple of ridges that merge ventrally with the pre/postspinal laminae. These ridges are topographically equivalent (and, possibly, serially homologue) to the spinozygapophyseal laminae of the caudal vertebrae (see below).

### Caudal vertebrae

**Fig 7:**
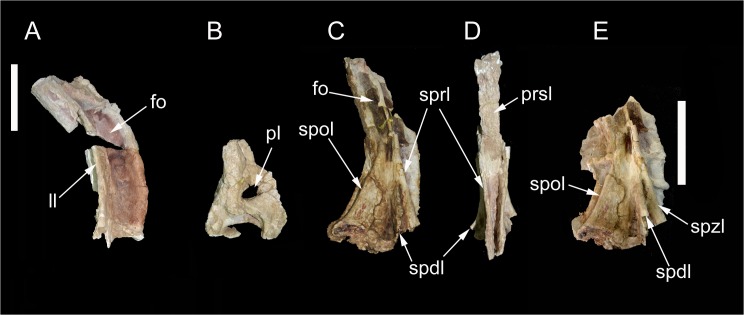
Partial proximal caudal vertebrae of *Tataouinea hannibalis*. Partial caudal neural spine 1 in right lateral view (A). Partial caudal centrum 1 in right lateral view (B). Partial neural arch 3 in right lateral (C) and proximal (D) views. Partial caudal neural arch 4 in right lateral view (E). Scale bars: A-D: 10 cm, E: 10 cm. Abbreviations: fo, fossa; ll, lateral lamina; pl, pleurocoel; prsl, prespinal lamina; spdl, spinodiapophyseal lamina; spol, spinopostzygapophyseal lamina; sprl, spinoprezygapophyseal lamina.

**Fig 8:**
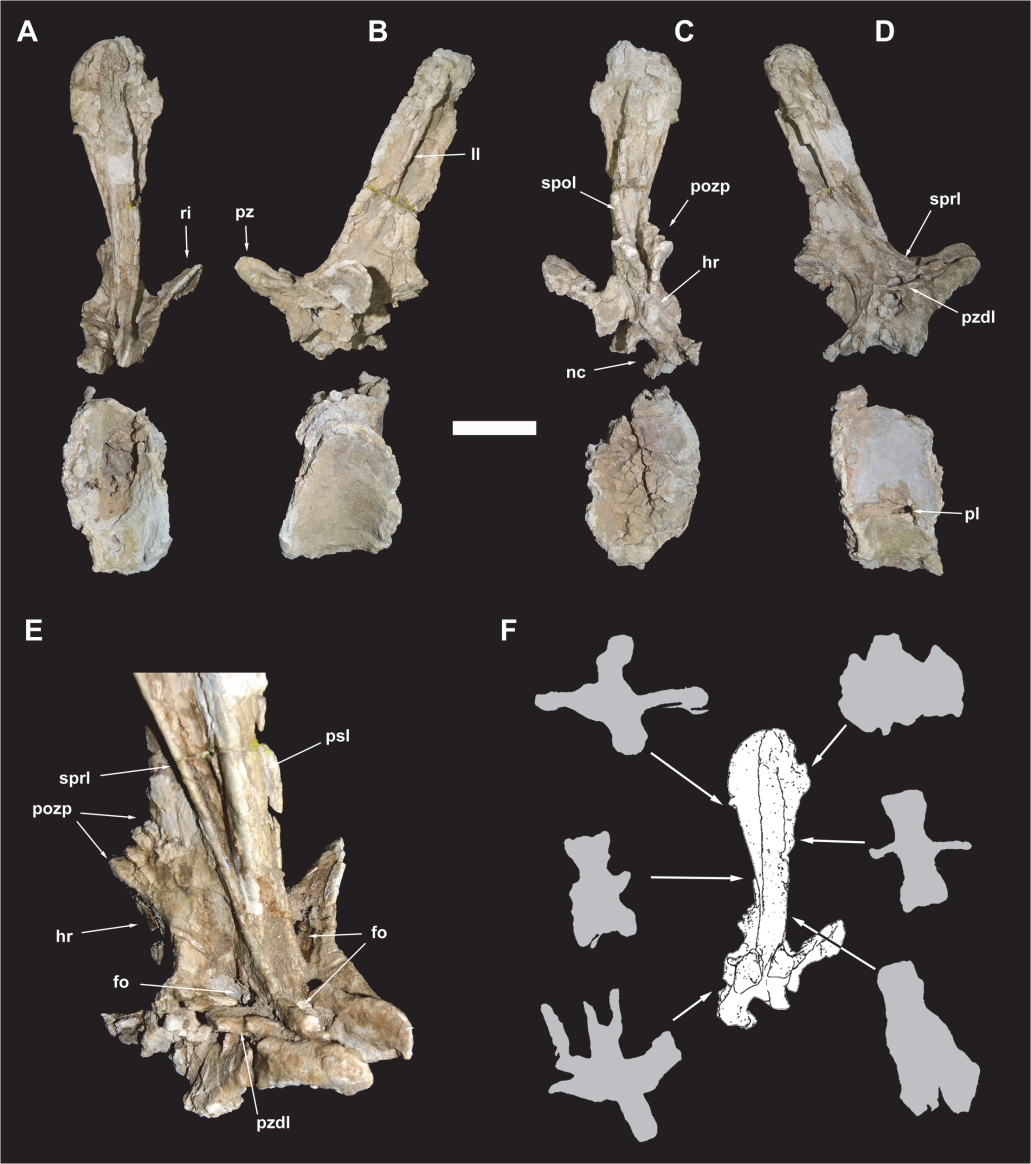
Caudal vertebra 5 of *Tataouinea hannibalis*. Vertebra in proximal (A), left lateral (B), distal (C), right lateral (D) views. Detail of neural arch in right proximodorsal view (D). Cross section shapes of neural arch (shown in proximal view) in six points indicated by arrows (proximal is bottom). Scale bar: 10 cm. Abbreviations: fo, fossa; hr, hyposphenal ridge; ll, lateral lamina; nc, neural canal; pl, pleurocoel; pozp, postzygapophysis pathology; psl, prespinal lamina; pz, prezygapophysis; pzdl, prezygodiapophyseal lamina; ri, rib; spol, spinopostzygapophyseal lamina; sprl, spinoprezygapophyseal lamina.

**Fig 9:**
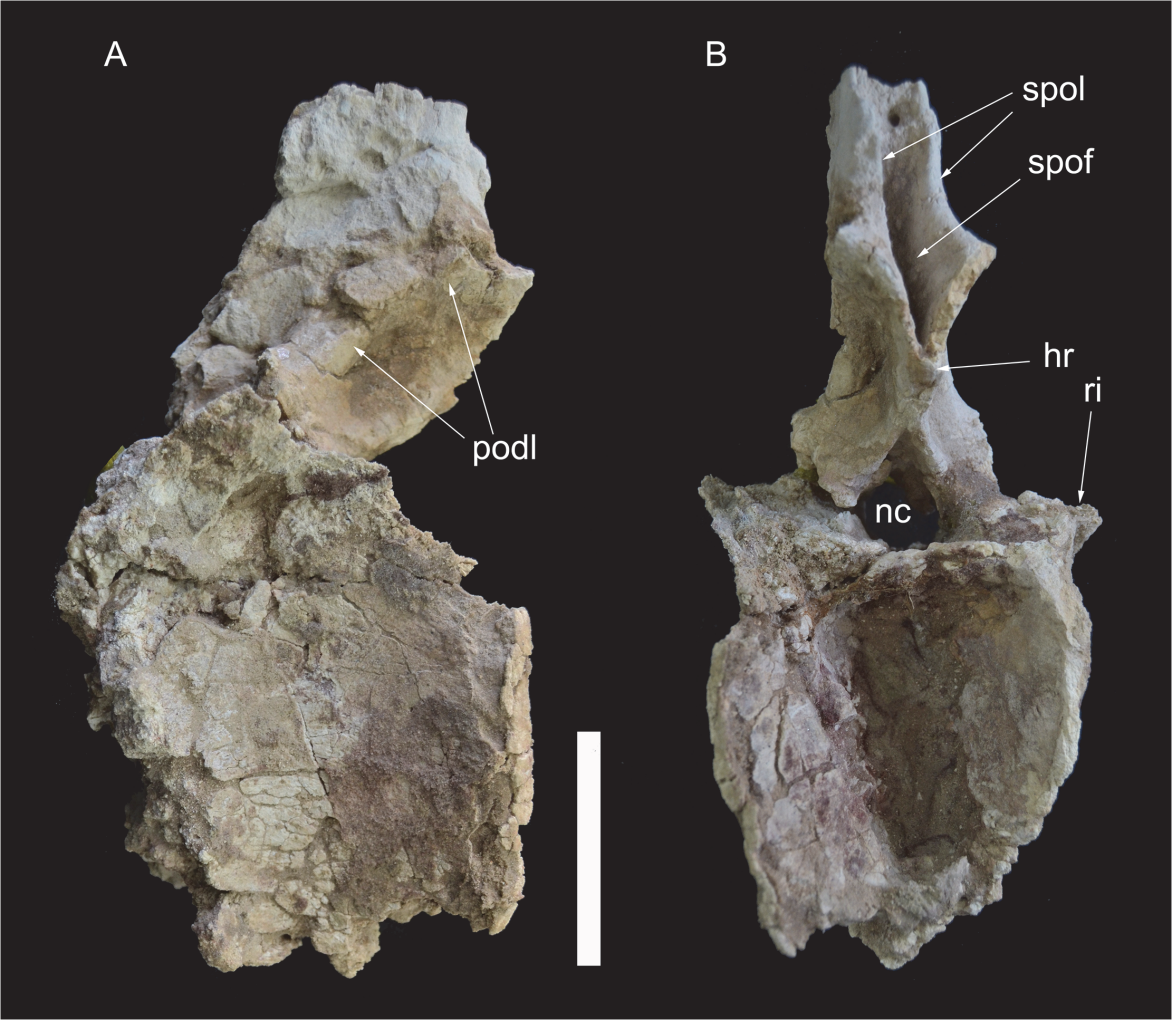
Caudal vertebra 6 of *Tataouinea hannibalis*. Vertebra in left lateral (A) and distal (B) views. Scale bar: 10 cm. Abbreviations: hr, hyposphenal ridge; nc, neural canal; podl, postzygodiapophyseal lamina; ri, rib; spof, spinopostzygapophyseal fossa; spol, spinopostzygapophyseal lamina.

**Fig 10:**
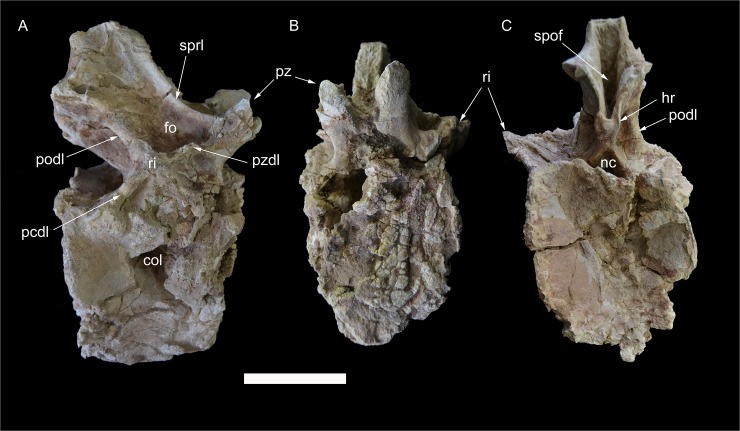
Caudal vertebra 7 of *Tataouinea hannibalis*. Vertebra in right lateral (A), proximal (B) and distal (C) views. Scale bar: 10 cm. Abbreviations: col, collapsed area; fo, fossa; hr, hyposphenal ridge; nc, neural canal; pcdl, posterior centrodiapophyseal lamina; podl, postzygodiapophyseal lamina; pz, prezygapophysis; pzdl, prezygodiapophyseal lamina; ri, rib; spof, spinopostzygapophyseal fossa; sprl, spinoprezygapophyseal lamina.

**Fig 11:**
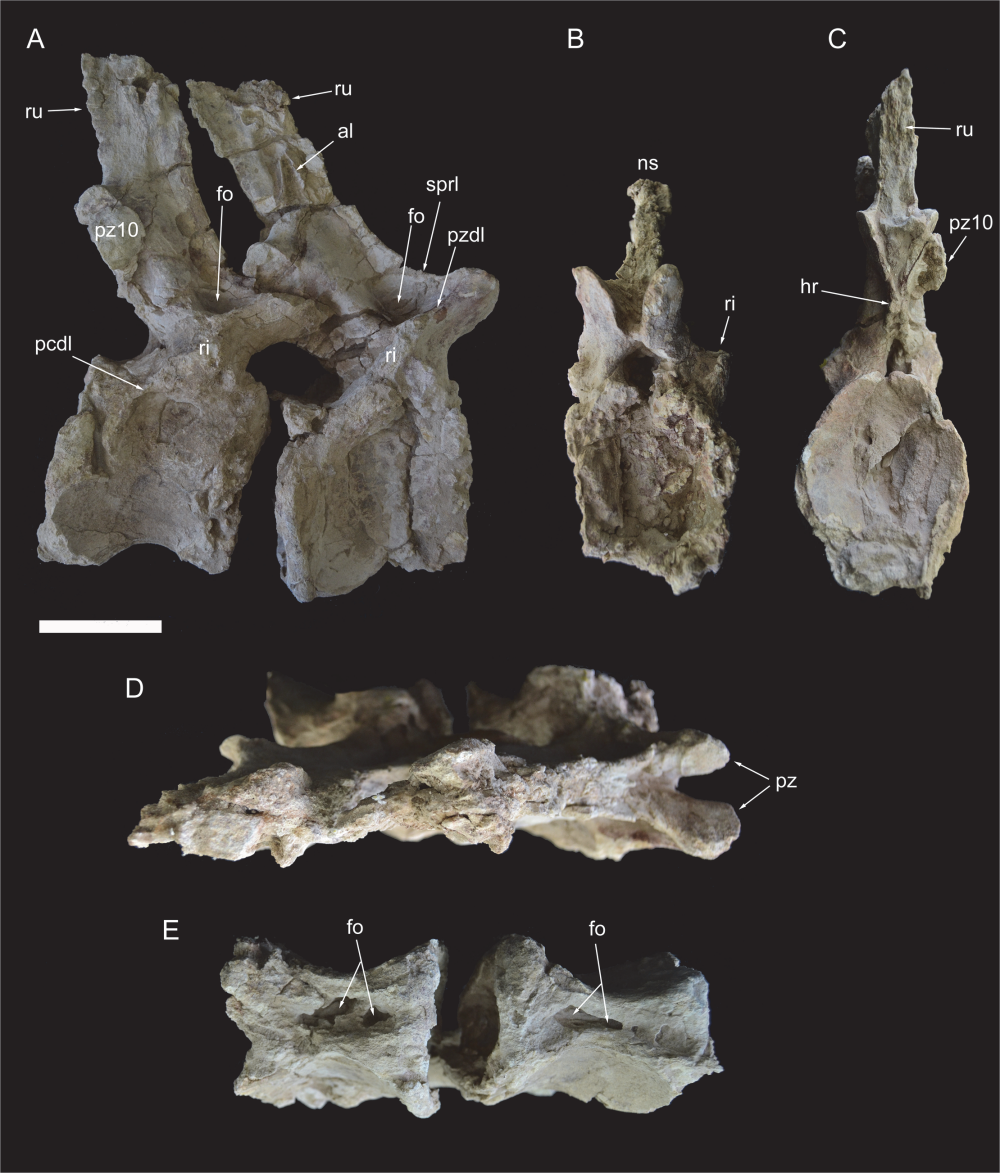
Caudal vertebrae 8 and 9 of *Tataouinea hannibalis*. Vertebrae in right lateral (A), proximal (B), distal (C), dorsal (D) and ventral (E) views. Scale bar: 10 cm. Abbreviations: al, accessory laminae; fo, fossa; hr, hyposphenal ridge; ns, neural spine; pcdl, posterior centrodiapophyseal lamina; pz, prezygapophysis; pz10, fragment of caudal 10 right prezygapophysis; pzdl, prezygodiapophyseal lamina; ri, ribs; ru, interspinal rugosity; sprl, spinoprezygapophyseal lamina.

**Fig 12:**
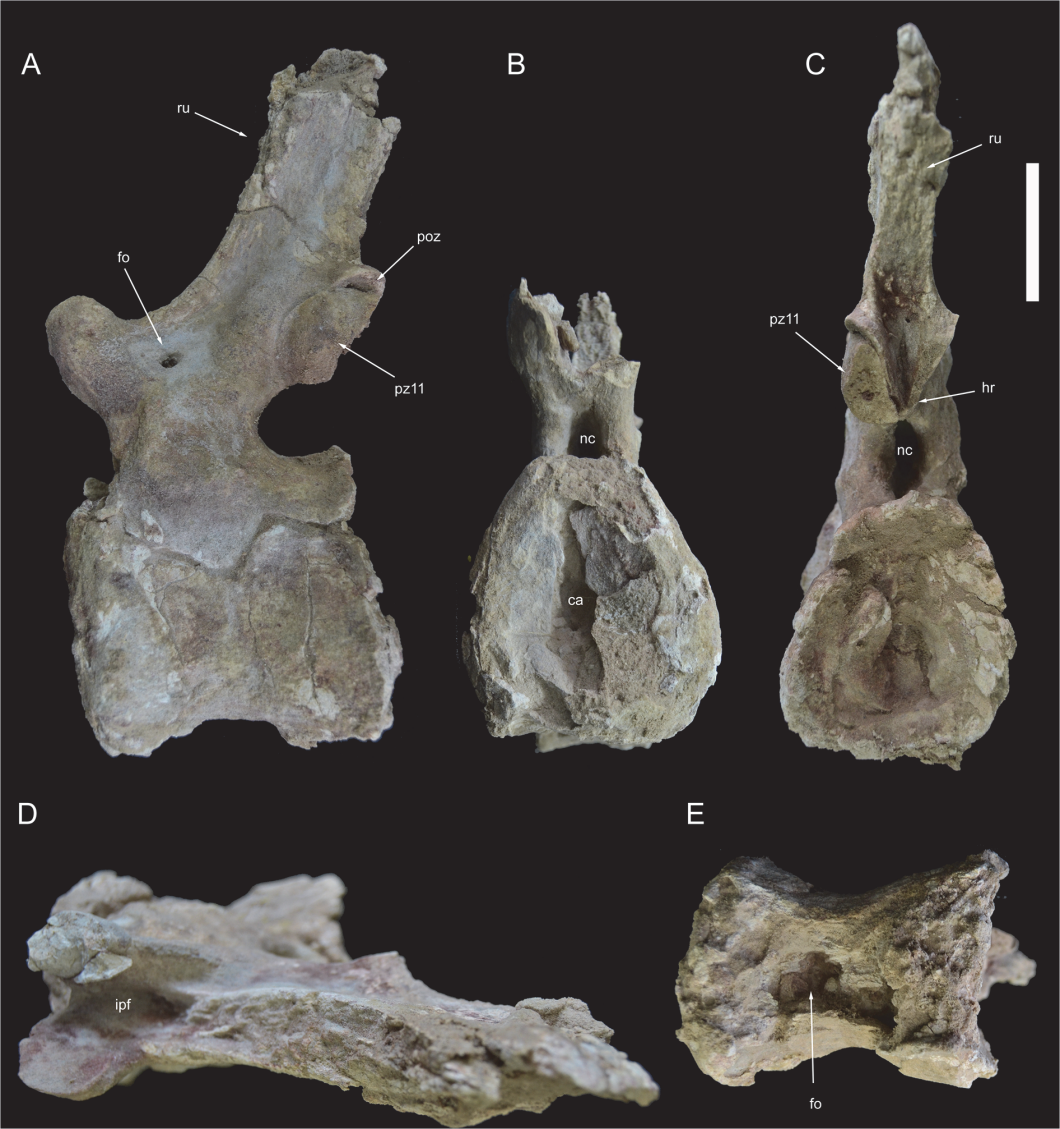
Caudal vertebra 10 of *Tataouinea hannibalis*. Vertebra in left lateral (A), proximal (B), distal (C), dorsal (D) and ventral (E) views. Scale bar: 10 cm. Abbreviations: fo, fossa; hr, hyposphenal ridge; ipf, interprezygapophyseal fossa; nc, neural canal; poz, postzygapophysis; pz11, fragment of caudal 11 left prezygapophysis; ru, rugosities.

**Fig 13:**
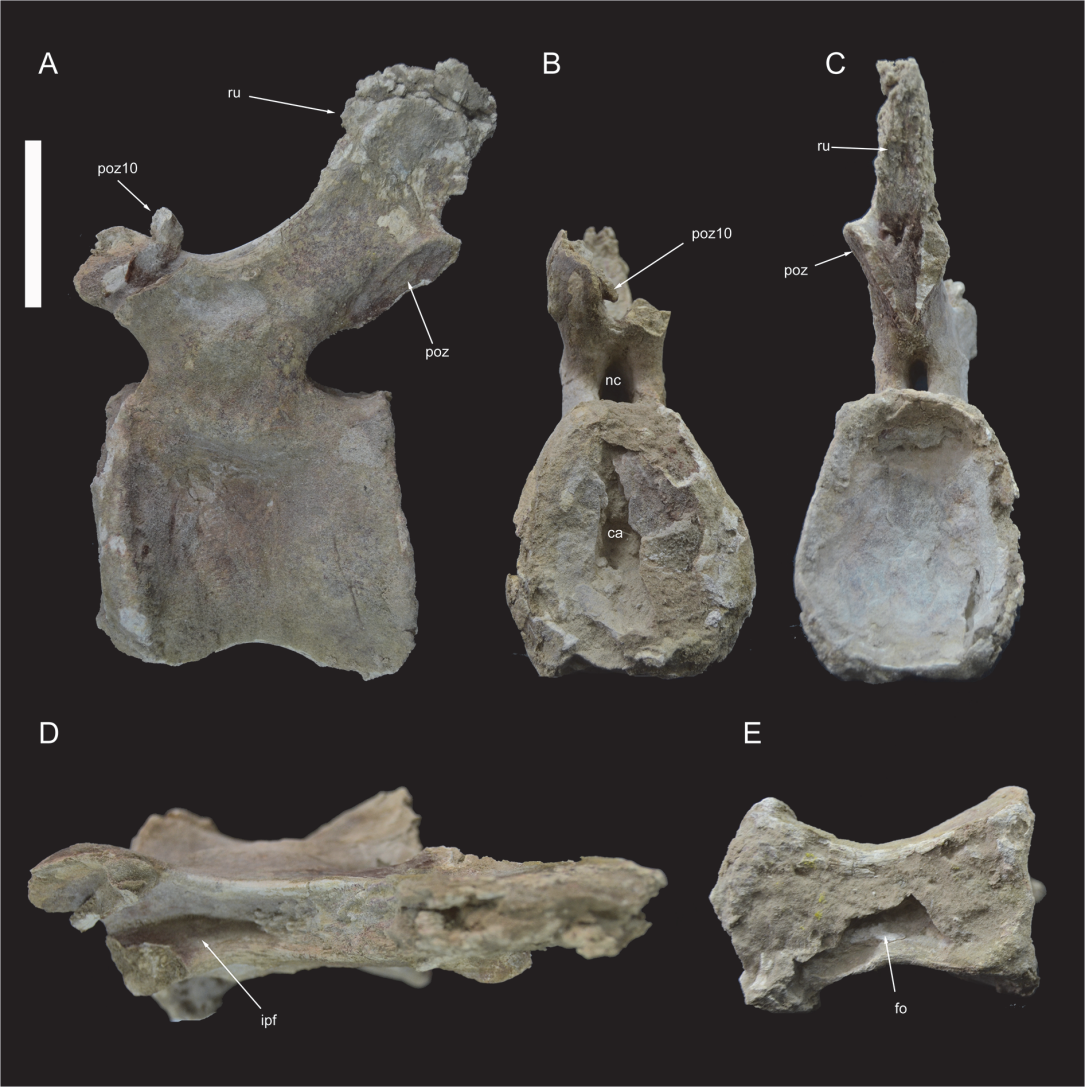
Caudal vertebra 11 of *Tataouinea hannibalis*. Vertebra in left lateral (A), proximal (B), distal (C), dorsal (D) and ventral (E) views. Scale bar: 10 cm. Abbreviation: ca, camerae; fo, fossa; ipf, interprezygapophyseal fossa; nc, neural canal; poz, postzygapophysis; poz10, fragment of caudal 10 right postzygapophysis; ru, rugosities.

**Fig 14:**
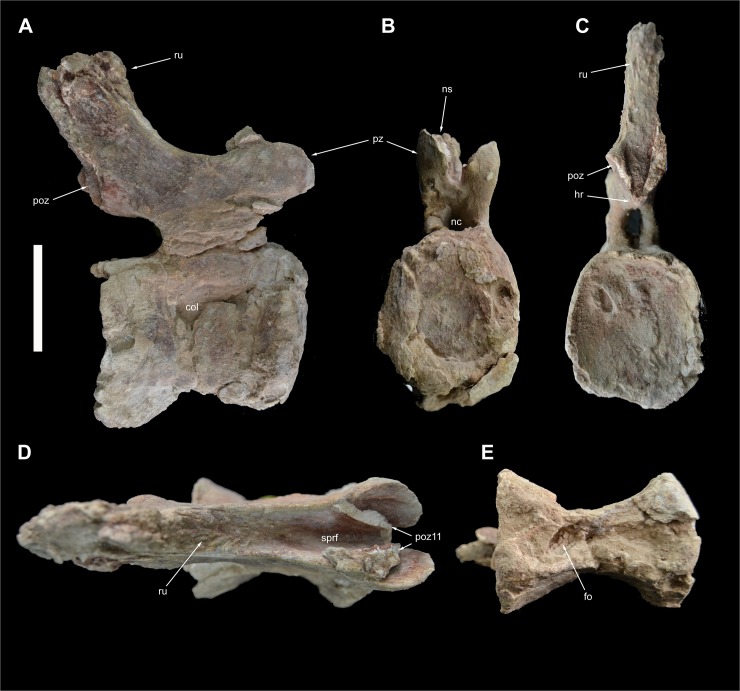
Caudal vertebra 12 of *Tataouinea hannibalis*. Vertebra in right lateral (A), proximal (B), distal (C), dorsal (D) and ventral (E) views. Scale bar: 10 cm. Abbreviation: fo, fossa; hr, hyposphenal ridge; nc, neural canal; ns, neural spine; poz, postzygapophysis; poz11, fragment of caudal 11 postzygapophysis; pz, prezygapophysis; ru, rugosities; sprf, spinoprezygapophyseal fossa.

**Fig 15:**
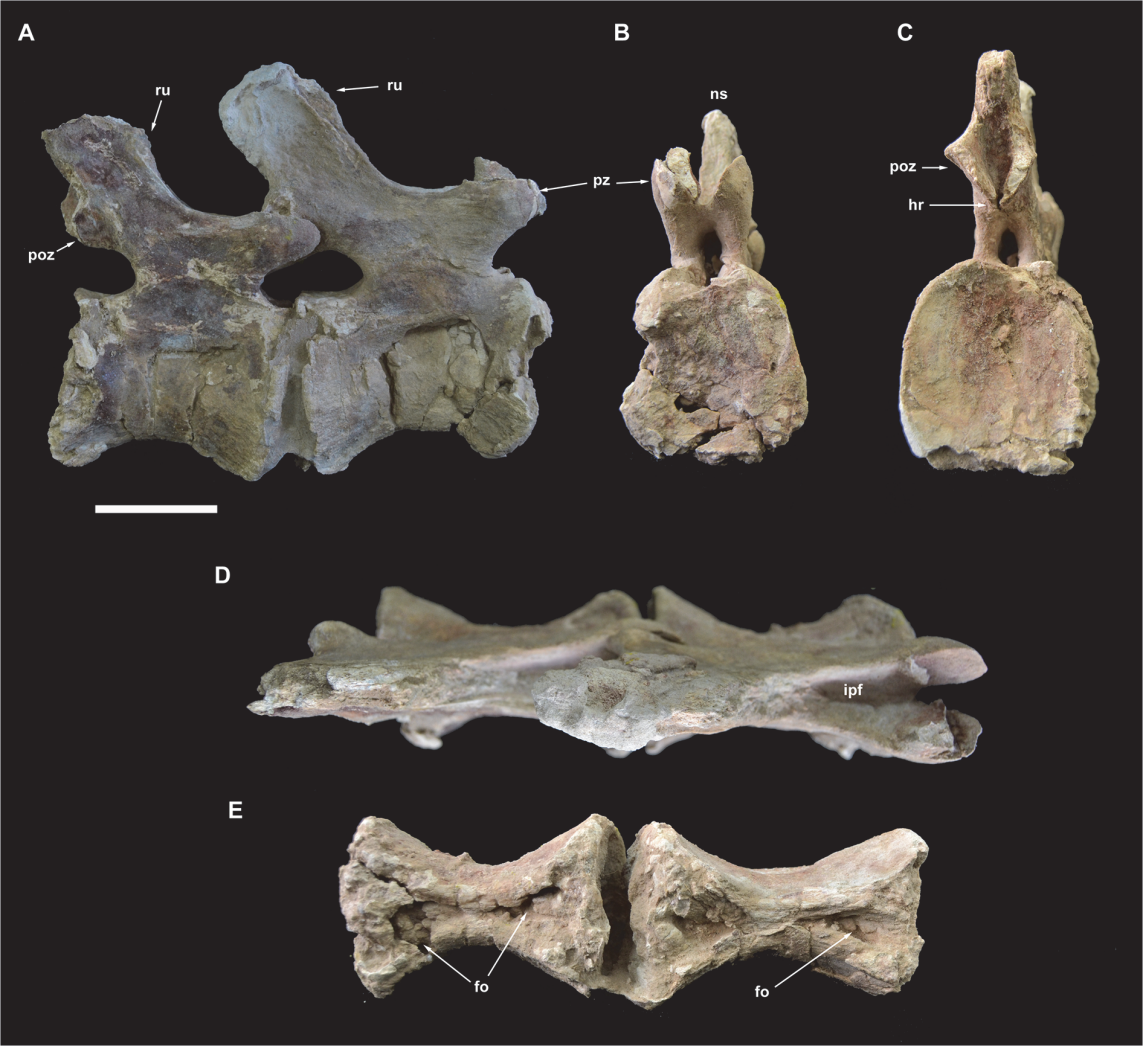
Caudal vertebrae 13 and 14 of *Tataouinea hannibalis*. Vertebrae in right lateral (A), proximal (B), distal (C), dorsal (D) and ventral (E) views. Scale bar: 10 cm. Abbreviations: fo, fossa; hr, hyposphenal ridge; ns, neural spine; poz, postzygapophysis; pz, prezygapophysis; ru, rugosities; sprf, spinoprezygapophyseal fossa.

**Fig 16:**
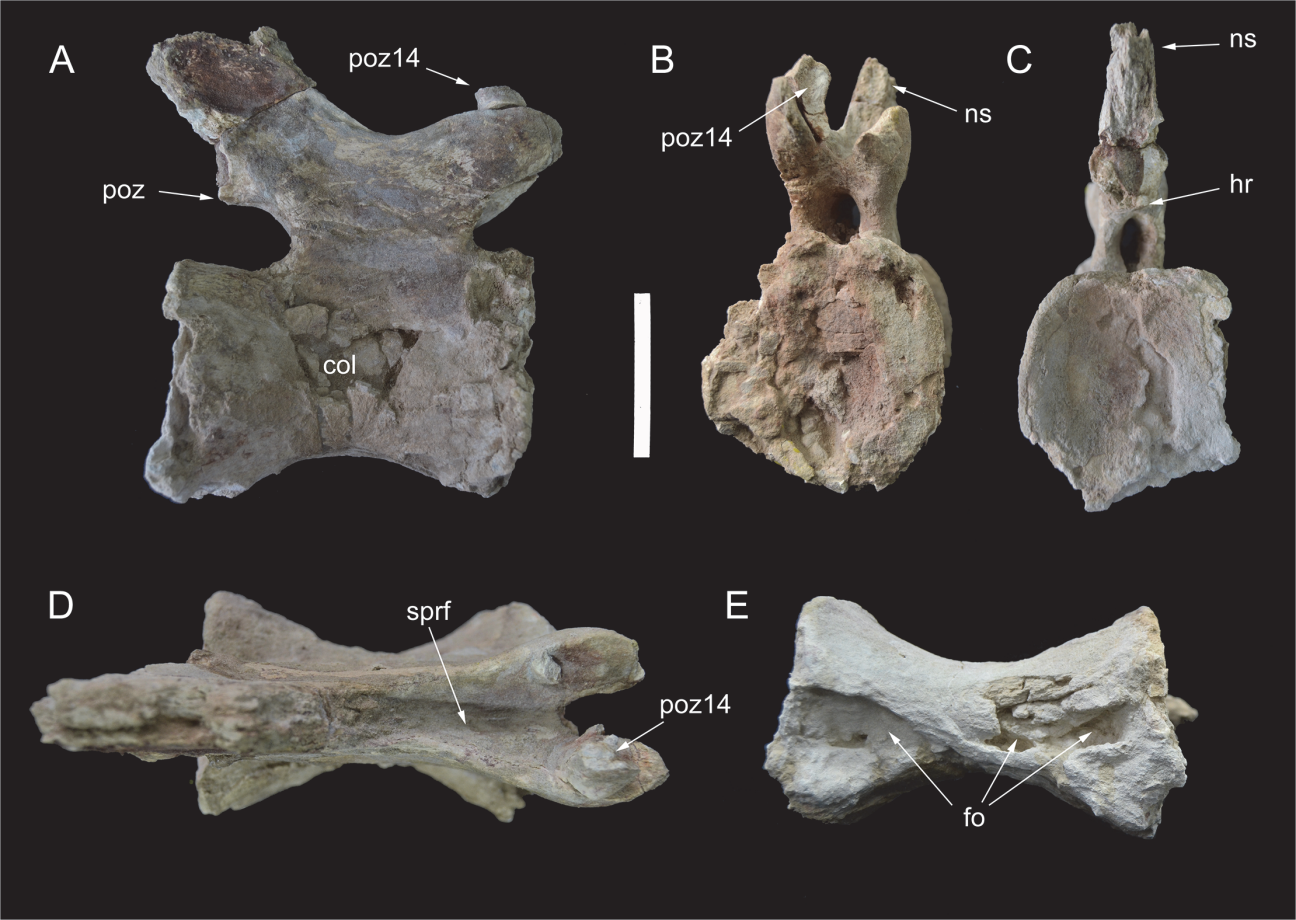
Caudal vertebra 15 of *Tataouionea hannibalis*. Vertebra in right lateral (A), proximal (B), distal (C), dorsal (D) and ventral (E) views. Scale bar: 10 cm. Abbreviations: col, collapsed area; fo, fossa; hr, hyposphenal ridge; ns, neural spine; poz, postzygapophysis; poz14, right caudal 14 postzygapophysis fragment; sprf, spinoprezygapophyseal fossa;.

**Fig 17:**
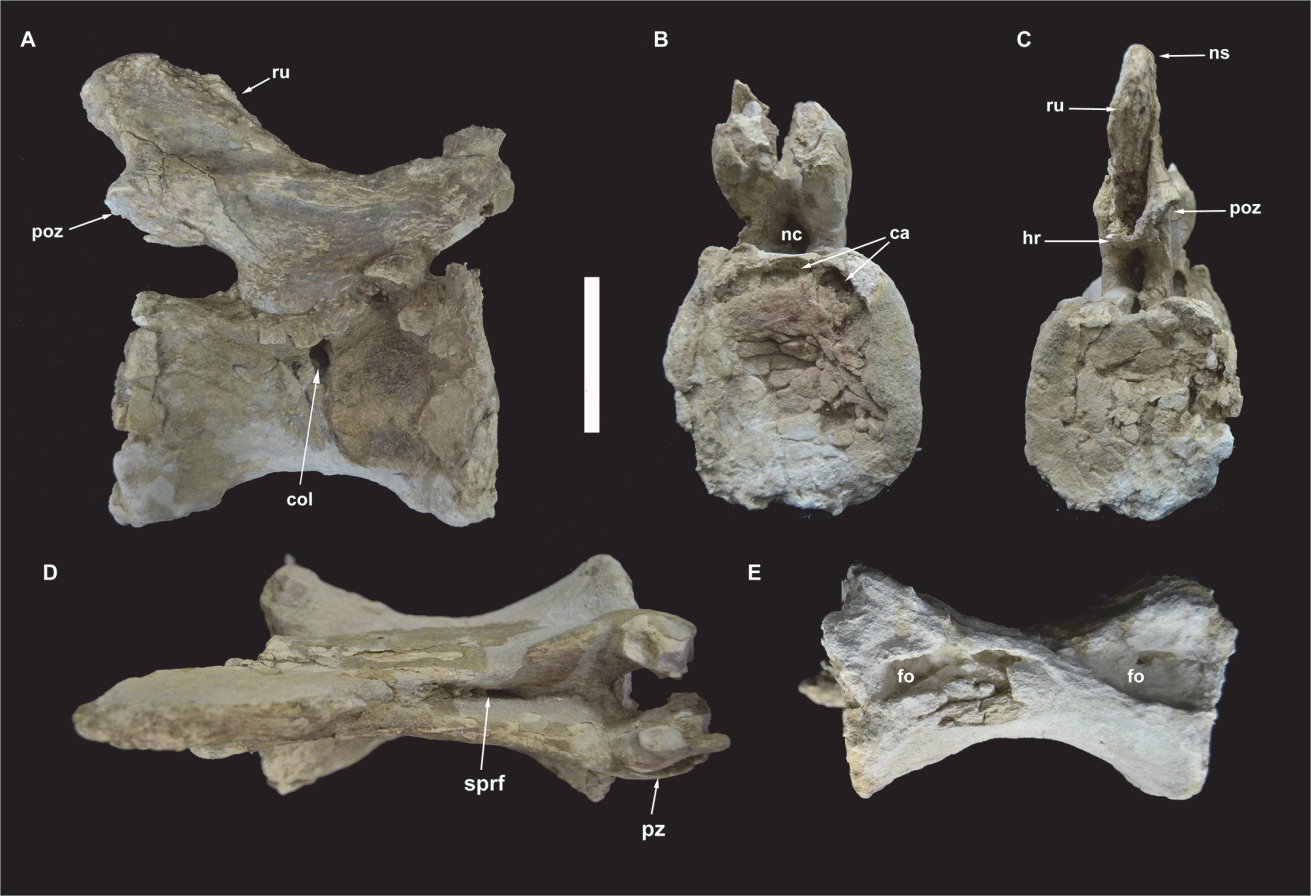
Caudal vertebra 16 of *Tataouinea hannibalis*. Vertebra in right lateral (A), proximal (B), distal (C), dorsal (D) and ventral (E) views. Scale bar: 10 cm. Abbreviations: ca, camerae; col, collapsed area; fo, fossae; hr, hyposphenal ridge; nc, neural canal; ns, neural spine; poz, postzygapophysis; pz, prezygapophysis; ru, rugosities.

**Fig 18:**
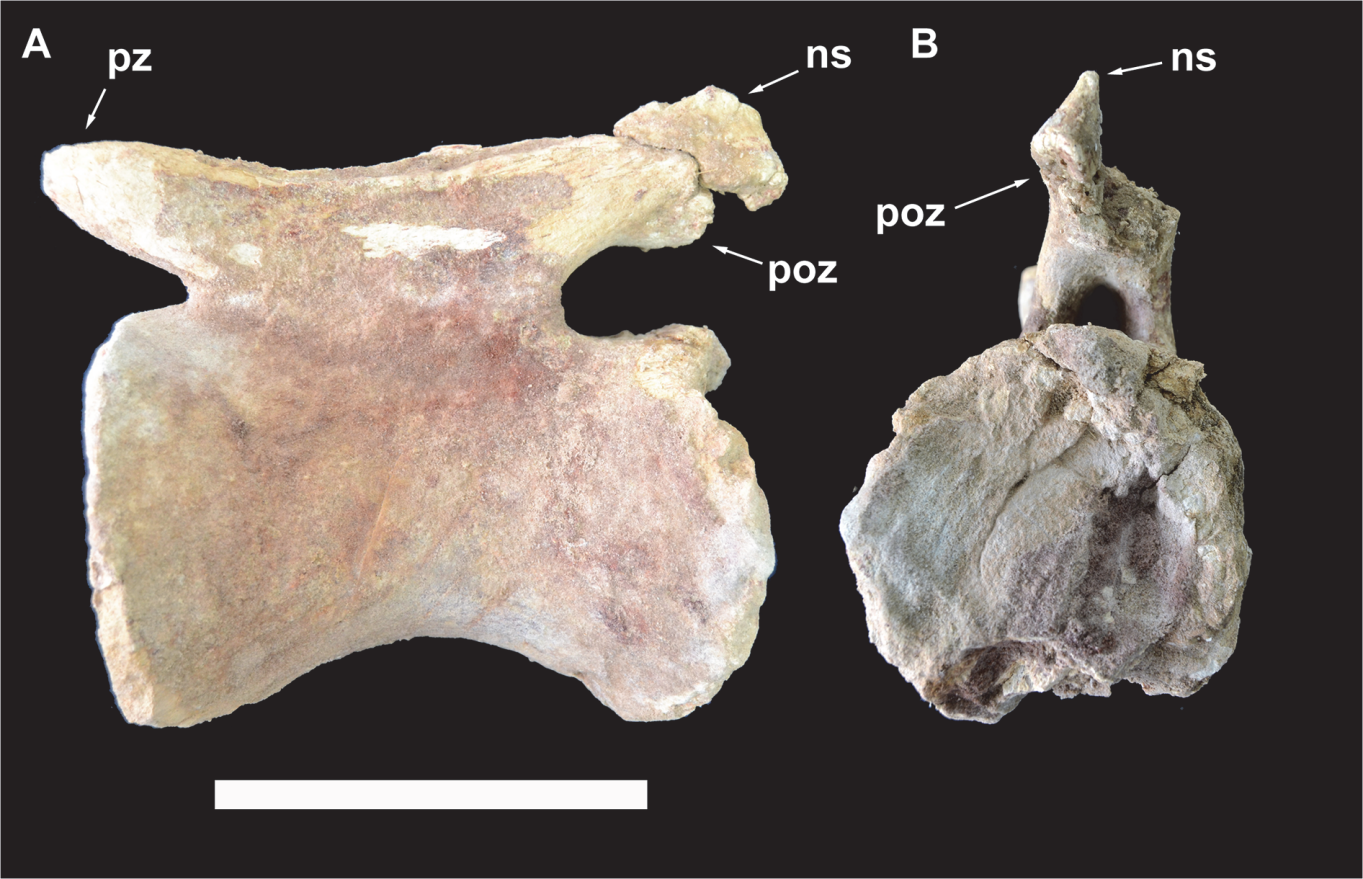
Caudal vertebra 17 of *Tataouinea hannibalis*. Vertebra in left lateral (A) and distal (B) views. Scale bar: 10 cm. Abbreviations: ns, neural spine; poz, postzygapophysis; pz, prezygapophysis.

The holotype of *Tataouinea hannibalis* includes the articulated series of the first seventeen caudal vertebrae. Fanti et al. [1] described only the first five proximal caudal vertebrae, the more distally placed vertebrae not yet uncovered at the time of submission of that study. Re-examination of the proximal caudal vertebrae preserved and comparison with photographs of the specimen *in situ* indicate that an additional centrum, although extremely fragmentary and considered part of the sacrum (Fig 3), is placed between caudal vertebrae 1 and 2 (numeration following [1]). We re-interpret that additional vertebra as the second caudal vertebra; and accordingly, caudal vertebrae 2 to 5 of [1] are reinterpreted here as caudal vertebrae 3 to 6. Caudal vertebrae 1 to 6 suffered important damage after collection (Figs 7 and 8), whereas the remaining vertebrae are exquisitely preserved and almost intact (Figs 9–18). All caudal vertebrae were found lying on the left lateral side, with the right side exposed and usually more weathered than the left one.

### Caudal 1 (ONM DT 8)

This vertebra included an almost complete centrum and a partial neural arch, missing most of the right lateral surface. The centrum is taller than long, with a markedly concave lateral surface (on both dorsoventral and proximodistal directions). The proximal intercentral facet was concave, and the distal facet more flattened. The ventral half of the lateral surface had collapsed internally. A large elliptical pneumatic foramen was present in the centre of the dorsal half of the lateral surface (Fig 7B). The neural arch was dorsoventrally elongate. The ribs were almost completely placed on the neural arch but were lost before the discovery of the specimen. Only the proximal part of the centrodiapophyseal laminae was present at the time of discovery. The zygapophyses were poorly preserved. The neural spine was dorsoventrally elongate, slightly dorsodistally inclined. The dorsodistal apex of the neural spine was placed at the level of the distal intercentral surface. The proximal margin of the neural spine was broadly convex and described a gentle curve in lateral view. Both pre- and postspinal laminae were badly preserved. The lateral surface of the neural spine was excavated by a couple of dorsoventrally oriented fossae, separated by a prominent lateral lamina (Fig 7A).

### Caudal 2 (ONM DT 19–22)

Only the partially preserved centrum of this vertebra was found. The centrum shows a quadrangular lateral surface pierced by an elliptical pneumatic opening.

### Caudal 3 (ONM DT 9)

Both the centrum and the neural arch of this vertebra were found. The centrum was taller than long and quadrangular in lateral view. The lateral surface of the centrum was badly preserved and the presence of a pneumatic foramen as in the adjacent vertebrae cannot be confirmed. The neural arch was almost complete. The ribs were placed almost completely on the neural arch, and projected dorsolaterally, exposing the ventral surface in lateral view. The proximal and distal margins of the rib were sub-parallel and inclined mostly laterally and slightly cranially in dorsoventral view. The prezygapophyses and the proximal surface of the neural spine are lost. The right lateral surface of the neural spine is partially preserved and shows a prominent spinoprezygapophyseal lamina directed dorsoventrally and joining the spinodiapophyseal lamina (Fig 7C and 7D). This lamina converges dorsally with the spinopostzygapophyseal lamina. The postspinal lamina is well developed and excavated laterally by a dorsoventrally oriented fossa.

### Caudal 4 (ONM DT 10)

This vertebra was well preserved, missing only the ribs and the dorsal end of the neural spine. The centrum was taller than long in lateral view and taller than wide in proximal/distal view. The intercentral facets were slightly concave. An elliptical foramen pierced the lateral surface of the centrum, at about mid-height. The preserved medial end of the rib was triangular in lateral view, with a broad ventral base. The zygapophyses were badly preserved. The neural spine was dorsoventrally elongate and inclined ventrodistally at about 30° relative to the longitudinal axis of the neural arch. The prezygodiapophyseal lamina was prominent and shelf-like. Both pre- and postspinal laminae were robust and excavated laterally by dorsoventrally oriented fossae. The prominent spinoprezygapophyseal lamina joins ventrally the spinodiapophyseal lamina forming the “lateral lamina” of the neural spine (Fig 7E). The spinopostzygapophyseal lamina was well developed and projected proximodorsally. Although the spinoprezygapophyseal and spinopostzygapophyseal laminae bounded a lateral triangular fossa, as in caudal 3, the bad preservation of the dorsal end of the neural spine prevented us for determining whether these laminae merged at their dorsal end.

### Caudal 5 (ONM DT 11)

The centrum is partially preserved (Fig 8A–8D), missing the right dorsal corner of the proximal facet, part of the left ventral corner of the distal surface and the left side of the ventral surface. The centrum is twice taller than long in lateral view, and twice taller than wide in proximal and distal views. The intercentral facets are elliptical, and both distinctly concave, with the proximal facet showing a more marked concavity than the distal. The lateral surfaces are longitudinally concave in dorsal view. The ventral surface shows a prominent ridge running along the ventrolateral margin of the right surface delimiting a central sulcus (the contralateral ridge on the left side is lost). On the left surface of the centrum, an elliptical pneumatic foramen opens just below the mid-height of the centrum. The pleurocoel is longer than tall. No foramen is present in the right surface of the centrum, an asymmetrical pattern of pneumatisation also present in the sacral vertebrae (see above) and in the ischia (see below). No ribs are present on the centrum, the former being entirely placed on the neural arch. Most of the neural arch is preserved: the left side of the pedicels, the left rib and the ventral part of the right rib are lost (Fig 8A–8E). The neural arch pedicels are longer than tall, and the neural canal is sub-oval in proximal view. The prezygapophyses are prominent and projected well proximal to the intercentral facet level. The postzygapophyses are slightly elevated relative to the prezygapophyses, and supported ventrally by a prominent hyposphenal process. The dorsal surface of the left postzygapophysis shows a “hinge-like” morphology, not present on the right postzygapophysis, and thus interpreted as probably pathological (Fig 8C and 8E). Two crests, oriented transversely and subparallel to the dorsodistal corner of the postzygapophysis, and two concavities, one between the two crests and the other between the distal crest and the dorsodistal corner of the postzygapophysis, form this feature. The neural spine is dorsoventrally elongate in lateral view, with straight proximal and distal margins inclined 30° dorsodistally relative to the vertical axis of the centrum, and a complex cross section geometry (Fig 8F). In proximal view, the neural spine is petal-shaped, with a distinctly lobate dorsal outline. A small lateral projection on the dorsal third of the neural spine is interpreted as homologue to the triangular process present in caudal vertebrae of other rebbachisaurines [e.g., 42]. The left rib is partially preserved, missing the ventral part. The dorsal component of the rib is wing-shaped in dorsal view and inclined laterodorsally at about 45° in proximal/distal view. The spinoprezygodiapophyseal fossae are deep and pierced in their middle by an elliptical foramen (Fig 8E). The prezygodiapophyseal, centroprezygapophyseal and centrodiapophyseal laminae are prominent and bound a triangular fossa (Fig 8E). The prespinal lamina is thick and hollowed laterally by drop-shaped depressions oriented dorsoventrally. These depressions are bounded laterally by the spinoprezygapophyseal laminae, the latter contacting the lateroventral end of the prespinal lamina. The floor of the spinoprezygapophyseal fossa is pierced by an elliptical foramen just proximally to the basal end of the prespinal lamina. The spinoprezygapophyseal laminae run laterodorsally and form the prominent lateral laminae of the neural spine. The postspinal lamina is thick and prominent as the prespinal lamina. The spinopostzygapophyseal laminae join the ventrolateral corner of the prespinal lamina and bound a deep interpostzygapophyseal fossa. The postzygodiapophyseal and the distal centrodiapophyseal laminae form the ventrodistal margin of the neural arch. These laminae and the hyposphenal ridge define a shallow elliptical fossa placed proximoventrally relative to the postzygapophyseal facet.

### Caudal 6 (ONM DT 37)

In this vertebra, both neural arch and centrum are well preserved and tightly sutured together (Fig 9). The proximal surface and part of the ventral surface of the centrum are mostly eroded away. The centrum is about 130% taller than long, and about 120% taller than wide. The distal intercentral facet is markedly concave, although this seems as partially a preservational artefact. The ventral half of the centrum is mediolaterally narrower than the dorsal, resulting in a trapezoid centrum with ventromedially directed lateral margins in distal view. The neural arch includes relatively low pedicels and a dorsally directed neural spine that is less elongated dorsally than the more proximal vertebrae. The base of the ribs is placed entirely on the neural arch, and extends proximodistally along most of the latter. The rest of the ribs is lost. The neural canal is round and its diameter is less than one fourth of the neural arch width. The prezygapophyses are lost. The postzygapophyses are elevated at about half the combined height of the neural arch and spine. The prespinal lamina is well developed, transversely robust, and includes the spinoprezygapophyseal laminae in its lateral component. The centrodiapophyseal laminae are poorly preserved. A robust hyposphenal ridge is directed dorsodistally and bifurcates to reach the postzygapophyses. The spinopostzygapophyseal laminae are directed dorsally, and bound a deep postspinal fossa floored by the hyposphenal process. A pair of hyposphenal postzygodiapophyseal fossae are present ventrolaterally to the postzygapophyses. The narrow prezygodiapophyseal lamina is horizontally directed. The postzygodiapophyseal lamina is more robust, and does not extend above the level of the postzygapophysis, neither forms a lateral lamina along the neural spine.

### Caudal 7 (ONM DT 38)

This vertebra is well preserved, lacking only the dorsal half of the neural spine and the lateral end of both ribs (Fig 10). The anterior half of the vertebra has suffered a more intense transversal compression than the posterior part, producing a partial dislocation of the neural arch, that is shifted onto the proximolateral corner of the right side of the centrum. The centrum is slightly taller than long in lateral view, taller than wide proximal view (in part due to transversal compression) and approximately as tall and wide in distal view. The proximal intercentral facet is flat, whereas the distal facet is distinctly concave. The right lateral surface of the centrum is depressed by a large irregular fossa, the depth of which is probably exaggerated by the vertebra deformation. No lateral fossa in present in the left side of the centrum. The ventral surface of the centrum is transversely narrow, sub-rectangular in ventral view, with parallel lateral sides that are projected ventrally delimiting a midline sulcus. The neurocentral suture is obliterated. The neural arch extends above the proximal two thirds of the centrum. The bases of the ribs are placed at the level of the neurocentral suture, and are inclined laterodorsally. Prominent distal centrodiapophyseal laminae bound the depressed dorsodistal surface of the centrum, although this may be a taphonomic artefact. The prezygapophyses are projected proximodorsally well beyond the level of the proximal intercentral surface. The prezygapophyses are widely separated and are not joined by a ventral interprezygapophyseal lamina. The postzygapophyses are placed more dorsally than the prezygapophyses. A prominent hyposphenal ridge joins the ventral base of the postzygapophyses and the dorsodistal margin of the neural canal. The preserved base of the neural spine is placed in the distal end of the neural arch. The neural spine is moderately thick. Both spinoprezygapophyseal and spinopostzygapophyseal laminae are present and well developed. The spinoprezygapophyseal laminae form the sharp lateral margins of the prespinal lamina. The spinopostzygapophyseal laminae bound laterally a deep postspinal fossa, the latter bounded ventrally by the hyposphenal ridge. The prezygodiapophyseal lamina is sharp. The prezygospinodiapophyseal fossae are deep. In the right prezygospinodiapophyseal fossa, an accessory ridge links the middle of the medial margin of the spinodiapophyseal lamina with the floor of the fossa. The prominent postzygodiapophyseal laminae are posteriorly concave and bound the shallow hyposphenal postzygodiapophyseal fossae.

### Caudal 8 (ONM DT 39)

This vertebra and caudal 9 are almost completely preserved, lacking the dorsal end of the neural spines (Fig 11). The two vertebrae were found tightly connected at the zygapophyses and were not separated after the discovery. In ONM DT 39, only the lateral end of the ribs is missing. The centrum is about as long as tall in lateral view and 4/3 taller than wide in proximal view. Both intercentral facets are elliptical in outline and concave, with raised lips along the margins. The ventral surface is hourglass shaped and bears a deep longitudinal pneumatic excavation. The lateral surfaces of the centrum lack pneumatic fossae. The neural arch extends above the proximal three quarters of the centrum. The ribs are entirely on the neural arch. Prominent proximal and distal centrodiapophyseal laminae are present and dorsally bound a distinct fossa. The neural spine is more than twice taller than long, placed above the distal end of the neural arch. The neural spine is quadrangular in lateral view and inclined dorsodistally. The prezygapophyses are widely spaced and project proximally beyond the intercentral facet. The prezygodiapophyseal and centroprezygapohyseal laminae are prominent and bound a shallow triangular fossa. The spinoprezygapophyseal laminae are sharp and run along the proximolateral margins of the neural spine. The prezygospinodiapophyseal fossa is deeply marked proximally, whereas its distal margin is indistinct from the lateral surface of the neural spine. The postzygapophyses are dorsally placed relative to the prezygapophyses, but most of their details are not visible as they are covered by the prezygapophyses of caudal vertebra 9. The postzygodiapophyseal lamina is low and robust. The ventral end of the spinopostzygapophyseal laminae bound a deep postspinal fossa restricted to just above the postzygapophyses. At mid-height on both lateral surfaces of the neural spine, a pair of short lateral laminae is present. These laminae may be interpreted as serially homologue to the more prominent lateral lamina present in the proximal caudal vertebrae.

### Caudal 9 (ONM DT 40)

This vertebra is almost completely preserved, lacking only the dorsal end of the neural spine, and is very similar in overall shape and proportions to caudal 8 (Fig 11). The main differences from the preceding vertebra are the presence of a depressed area on the ventral half of the left lateral surface of the centrum, the less prominent spinozygapophyseal and centrodiapophyseal laminae, and the poor development of the pair of accessory laminae on the lateral surface of the neural spine.

### Caudal 10 (ONM DT 41)

This vertebra is almost completely preserved, lacking the dorsal end of the neural spine (Fig 12). The centrum is longer than tall in lateral view and about as wide as tall in proximal view. Both intercentral facets are distinctly concave, with thickened rims. The ventral surface of the centrum is hourglass shaped and excavated by a deep fossa housing a pair of pneumatic openings. The lateral surfaces of the centrum are moderately compressed transversely. The neural arch extend along three fourths of the dorsal surface of the centrum. The neural arch pedicels are proportionally narrower transversely and taller than in the more proximal vertebrae. The neural canal is elliptical, taller than wide. The ribs are reduced to slightly raised tuberosities. The centrodiapophyseal lamination is, accordingly to rib reduction, very poorly developed. The prezygapophyses are short and inclined dorsally, not projected proximally beyond the centrum intercentral facet, and define a “V”-shaped cleft in proximal view. The spinoprezygapophyseal laminae are moderately developeded and bound dorsally a shallow prezygospinodiapophyseal fossa, more prominent on the left side. The postzygapophyses are closely placed medially, being less prominent than in more proximal vertebrae, and are supported ventrally by a small hyposphenal ridge. The neural spine is transversely narrower than in more proximal vertebrae, and is slightly inclined dorsodistally. The lateral surfaces of the neural spine shows a slightly developed lateral lamination oriented dorsoventrally. The anterior end of the left prezygapophysis of caudal 12 is tightly attached to the left postzygapophysis.

### Caudal 11 (ONM DT 42)

This vertebra is almost completely preserved (Fig 13), a fragment of the left prezygapophysis is attached to caudal 10 (Fig 12A and 12C). The centrum is longer than tall and taller than wide in proximal view. The intercentral facets are elliptical and distinctly concave (the distal concavity is more pronounced than the proximal). The ventral surface is hourglass shaped with a shallow ventral sulcus in the middle. Both lateral surfaces of the centrum are transversely concave and slightly overlapped laterodorsally by the neural arch. The right side of the lateral surface of the centrum has collapsed internally, suggesting a hollow interior of the centrum. The neural arch appears as transversely compressed and long about half of the dorsal surface of the centrum, and displaced slightly proximally relative to the centrum mid-length. The neural arch pedicels are transversely compressed and the neural canal is taller than wide. The prezygapophyses slightly overhang the proximal intercentral surface. The ribs are extremely reduced as small rugosities oriented proximodistally below the level of the prezygapophyses. Most of the neural arch lamination observed in more proximal vertebrae is absent. The subrectangular neural spine is lower than in more proximal vertebrae, placed at the dorsodistal end of the neural arch and poorly inclined distally. The spinoprezygapophyseal laminae are sharply elevated dorsally and bound a deep and narrow sulcus between the neural spine and the prezygapophyseal bases. The postzygapophyses are closely appressed, and projected laterodistally at mid-height of the distal margin of the neural spine. The hyposphenal ridge is less prominent than in more proximal caudal vertebrae.

### Caudal 12 (ONM DT 43)

The elongate centrum (about 125% longer than tall) of this completely preserved vertebra is taller than wide in proximal view (Fig 14). The intercentral facets are elliptical and moderately concave. The centrum is markedly compressed transversely with both lateral surfaces collapsed internally just ventral to the neurocentral contact. It is unclear whether the collapsed areas represents pneumatic features, although comparison with the following three vertebrae (see below) suggests that the collapsed areas housed pneumatic depressions in origins. The ventral surface of the centrum is hourglass-shaped, and bears a shallow mid-line sulcus bearing a distal elliptical fossa. The neural arch pedicels are relatively low and the neural canal elliptical, taller than wide. The ribs are extremely reduced as very low rugosities. No diapophyseal lamination is present. The prezygapophyses are proximally directed and do not project beyond the intercentral surface. The sharp spinoprezygapophyseal laminae bound a distinct fossa on the dorsal surface of the neural arch. The postzygapophyses are placed at about the same dorsoventral level as the prezygapophyses. The hyposphenal ridge is narrow and bounds ventrally a triangular interpostzygapophyseal fossa. The neural spine is quadrangular, taller than ventrally long and slightly inclined dorsodistally.

### Caudal 13 (ONM DT 44)

This almost completely preserved vertebra is tightly attached to caudal 14 at the level of the postzygapophyses (Fig 15). The centrum is transversely compressed with the right surface collapsed internally. The centrum is 133% longer than proximally tall, and about as tall as wide in proximal view. The proximal intercentral facet is trapezoidal, wider ventrally. The distal intercentral facet is mostly covered by caudal 14. Both intercentral facets are moderately concave. The ventral surface of the centum is hourglass-shaped, bearing two proximodistally aligned drop-shaped depressions. The left lateral surface bears an elliptical depression placed in the distal half of the surface. The corresponding area in the right lateral surface lacks a depression. Nevertheless, the collapsed proximal half of the right lateral surface may indicate the presence, in life, of a lateral depression. The low neural arch bears proximally directed prezygapophyses not surpassing the level of the intercentral facet, and postzygapophyses placed at the same level of the prezygapophyses. A deep and narrow spinoprezygapophyseal fossa is bounded by sharp spinoprezygapophyseal laminae. The postzygapophyses are joined medioventrally by a small hyposphene-like projection. The ribs are absent. The neural spine is slightly taller than long and moderately inclined dorsodistally. Neural arch lamination is limited to both spinopre- and spinopostzygapophyseal laminae. Prespinal and postspinal laminae are restricted to the apical half of the spine and scarred by a rugose pattern.

### Caudal 14 (ONM DT 45)

This vertebra is very similar in overall shape and preservation to caudal 13, differing in the slightly shorter centrum, the better preservation of the centrum lateral surfaces, and the lower neural spine that is longer than tall (Fig 15). The most interesting feature of vertebra 14 is the presence of distinct fossae in the lateral surfaces of the centrum. The right surface of the centrum bears a narrower and slit-like depression, whereas the fossa on the lateral surface is clearly elliptical, proximodistally elongate, with distinct margins, similar to the pneumatic foramina present in the proximalmost caudal vertebrae. The distal fragment of the right postzygapophysis is attached to the corresponding prezygapophysis of caudal 15.

### Caudal 15 and 16 (ONM DT 46 and 47)

These completely preserved vertebrae show elongate centra (with the length to proximal height ratio of about 135–140%) with elliptical intercentral facets that are as wide as tall (Figs 16 and 17). In overall shape and proportions, these vertebrae are very similar to caudal 14. The ventral surfaces of the centra are hourglass-shaped and house shallow depressions. In both vertebrae, the left lateral surface of the centrum is excavated by a shallow depression, whereas the right lateral surface is collapsed internally and partially crushed. Cracks on the proximal intercentral facet of caudal 16 shows that the internal pneumatisation pattern is camerate. The prominent prezygapophyses are projected proximally beyond the level of the intercentral facet, and are linked to the neural spine by sharp laminae bounding a distinct interprezygapophyseal fossa. The postzygapophyses are reduced and joined ventromedially by hyposphene-like laminae. The neural spines are lower than in more proximalvertebrae and inclined dorsodistally.

### Caudal 17 (ONM DT 48)

This is the posteriormost preserved caudal vertebra (Fig 18). The vertebra is almost complete, lacking the right postzygapophysis and the tip of the neural spine. The centrum is elongate (about 150% longer than proximally tall), with roughly rounded intercentral facets that are as wide as tall. The right lateral surface of the centrum is collapsed at the neurocentral junction. The ventral surface of the centrum is transversely constricted but lacks the distinct fossae present in the more proximal vertebrae. The left lateral surface is undeformed and lacks any excavation or depression. Both zygapophyses and spinoprezygapophyseal laminae are less prominent than in more proximal vertebrae. The hyposphene is poorly preserved, and appears as a shallow lamina below the postzygapophyses.

### Ilium

**Fig 19:**
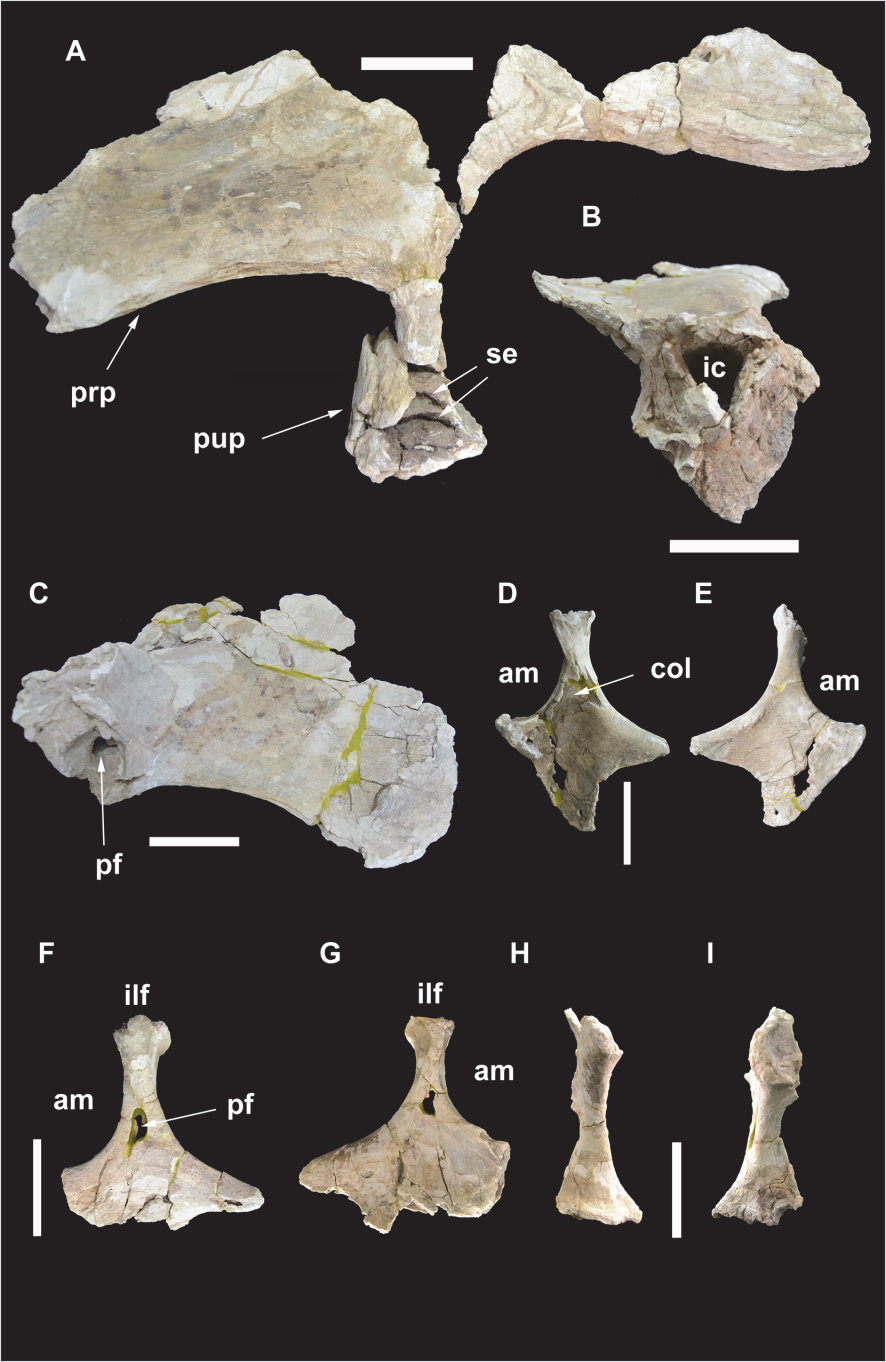
Pelvic elements of *Tataouinea hannibalis*. Left ilium in lateral view (A). Left pubic peduncle in distal/ventral view (B). Right preacetabular process of ilium in lateral view (C). Right ischium in medial (D) and lateral (E) views. Left ischium in lateral (F) and medial (G) views. Right ischium in proximal (acetabular) view (H). Left ischium in proximal (acetabular) view (I). Scale bar: 10 cm. Abbreviations: am, acetabular margin; col, collapsed area; ic, internal chamber; ilf, iliac facet; pf, pneumatic foramen; prp, preacetabular process; pup, pubic peduncle; se, septa.

Both iliac blades are partially preserved (ONM DT 3, 4; Fig 19A–19C). The dorsal margin of the bones, the postacetabular processes and most of the ischial peduncles are lost. The preacetabular processes are craniocaudally elongate, and flared laterally. In lateroventral view, the cranioventral corner of the preacetabular blade is gently acuminate, with a rounded craniodorsal margin and a slightly concave ventrolateral margin. The cranioventral corner of the lateral surface is rugose and scarred by an irregular series of low bumps. Most of the lateral surface of the blade is uniformly flat, showing a moderate longitudinal convexity toward the ventral margin, where the lateral surface shifts into the ventral surface. The preacetabular blade is internally hollow. A channel is exposed in the broken region craniodorsal to the pubic peduncle. It leads to an internal chamber on the iliac blade. The large size of the exposed channel and the extensive cavitation of the preacetabular blade suggest a pneumatic origin of the feature. It is unclear whether this channel opened externally through a foramen at the level of the damaged area. The pubic peduncle is massive, projected laterally relative to the ventrolateral surface of the preacetabular process, and describing with the latter a wide concavity. In ventral view, the pubic peduncle is “D”-shaped, with the straight caudal margin oriented mediolaterally and the cranial margin broadly rounded. The ventral end of the pubic peduncle is badly eroded, showing the internal pneumatisation composed of large chambers separated by dorsoventrally narrow septa oriented horizontally.

### Ischium

**Fig 20:**
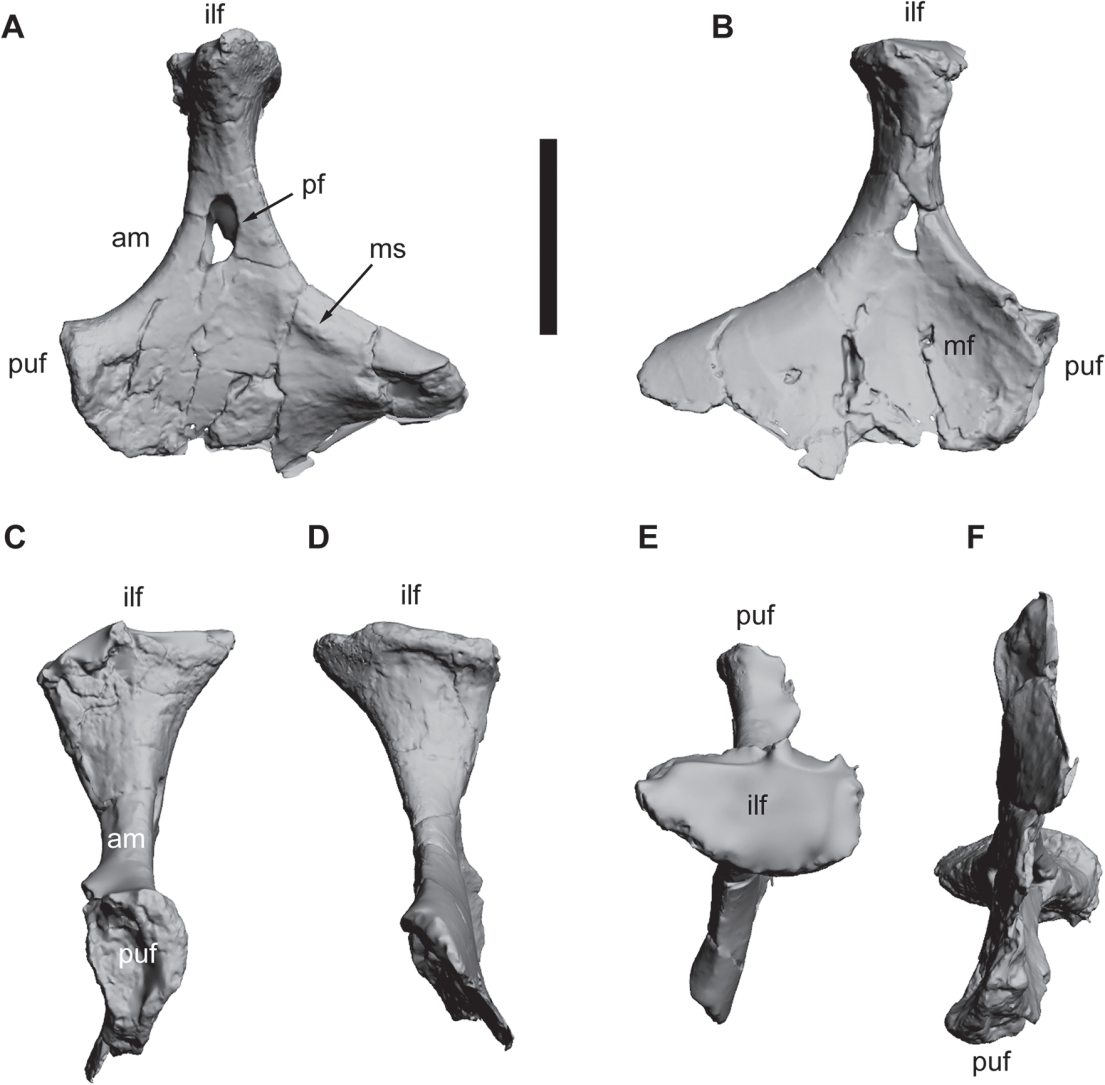
Left ischium of *Tataouinea hannibalis*. Ischium in lateral (A), medial (B), cranial (C), caudal (D), proximal/dorsal (E), distal/ventral (F) views. Scale bar: 10 cm. Abbreviations: am, acetabular margin; ilf, iliac facet; mf, medial fossa; ms, muscle scar; pf, pneumatic foramen; puf, pubic facet.

The proximal half of both ischia (ONM DT 1, 2) were found articulated with the iliac blades. The shaft distal to the pubic peduncle is lost in both ischia (Figs 19D–19I and 20). The two bones differ in the preservation of their extremities and in the degree of mediolateral deformation. In the left ischium, the iliac peduncle is almost complete, whereas in the right ischium the same peduncle lacks the cranial part of the proximal surface. In the right ischium, parts of both iliac and pubic peduncles are crushed and have been collapsed internally. The pubic peduncle in the right ischium is more complete distally than in the left ischium. Nevertheless, combining the information from the two bones, most of the morphology of the ischium, with the exclusion of the distal shaft, is available. The ischia are roughly quadrangular in mediolateral view, with a broadly concave acetabular margin, a dorsoventrally deep pubic facet, a gently concave caudodorsal margin. The acetabular margin is hourglass shaped in proximal view, expanded transversely toward both iliac and pubic peduncles and constricted at mid-length. The ischial body (the preserved part of the bone excluding the iliac peduncle and the pubic facet) is flattened and laminar distal to the acetabular margin. The lateral surface of the ischial body is flat to very gently convex toward the pubic facet. A distinct tuberosity is present on the dorsal margin of the lateral surface just distal to the iliac peduncle base. The medial surface of the ischial body is excavated by a depressed area between the iliac peduncle and the pubic facet. This depression is bounded craniodorsally by the thickened acetabular margin. In cranial view, the pubic facet is triangular, wider dorsally (the acetabular margin) and laminar ventrally. The iliac peduncle is proximodistally elongate and slender in mediolateral view, with the proximodistal axis inclined slightly cranially relative to a line tangential to both acetabular and caudodorsal margins. The iliac peduncle is slightly constricted at mid-height and expanded both craniocaudally and transversely approaching the ilium. The caudal margin of the iliac facet bears a distinct lip that overhangs the shaft of the peduncle. In proximal view, the iliac facet is elliptical, about 170% wider than long. In cranio/caudal view, the medial margin of the iliac peduncle is straight, whereas the lateral margin is gently concave and directed proximomedially. The most interesting feature of the ischia is the pneumatisation [1]. Both ischial bodies are hollowed internally by a chamber with smooth inner surfaces, each reaching the preserved distal end, although the two bones differ in the degree of preservation of these features. The lateral surface of the left ischium bears a large pneumatic foramen at the level of the distal half of the iliac peduncle. The medial surface of the bone is perforated by a smaller aperture, although it is unclear whether it represents a natural pneumatic foramen or the result of post-mortem collapse of the medial surface of the internal chamber. The lateral foramen in the left ischium is clearly a pneumatic feature, as it shows a defined margin with a regular elliptical outline (damaged on its anteroventral corner) comparable, in shape and proportion, to the pneumatic foramina present in the vertebrae, and leads to the internal chamber of the bone. Although the medial surface is internally collapsed in the right ischium, the lateral surface of the latter lacks a perforation, indicating that the extent of ischial pneumatisation was asymmetrically developed.

## Phylogenetic and Palaeogeographic Analyses

We tested the phylogenetic affinities of *Tataouinea* entering an Operational Taxonomic Unit (OTU) based on the Tunisian taxon in an updated version of the data set of [44] focusing on sauropods. As the diplodocoid affinities of *Tataouinea* are well supported and based on several synapomorphies from both caudal vertebrae and pelvis [1], we removed *a priori* most of the non-diplodocoid OTUs from the original data set, and retained a subset of taxa sampling the morphological diversity among eusauropods. We added five additional characters to the data set of [44], and derived from [41], [43], and [40]. The characters were set with equal weight and all multistate characters as unordered (non-additive). *Rebbachisaurus* was re-scored based on the recent revision of the taxon by [40]. We also added the recently named Patagonian rebbachisaurid *Katepensaurus* [43], not included before in a quantitative phylogenetic analysis. Trees were rooted on the basal eusauropod *Shunosaurus*. This dataset (29 OTUs vs 346 characters) was analysed under both parsimony and Bayesian inference, the latter integrating simultaneously morphological and stratigraphic data following the method discussed by [45] and [46]. Among the 346 included characters, 90 characters are constant, and 55 characters are autapomorphies of the included taxa, as Bayesian analysis requires the sampling of not solely synapomorphies, but also autapomorphies of terminal branches and constant characters [46]. Parsimony analyses were performed using the Hennig Society version of TNT [47]. The analyses followed two steps: 1) 100 driven searches using the “New Technology analyse” set in TNT with default parameters, followed by 2) a “Traditional Search” analysis exploring the tree islands found by the first run. Nodal support (Decay Index) was calculated performing 1000 “Traditional Search” analyses and saving all trees up to ten steps longer than the shortest topologies. Bayesian analyses were performed using BEAST vers. 1.7 [48] implementing Markov-Chain Monte Carlo Bayesian methods for estimating phylogeny, and using the Lewis’s Markov model [49] for discrete character evolution, as it accommodates variability in rates of evolution among characters (using the gamma distribution) and across lineages (using relaxed clocks). All characters were treated as a single partition. Stratigraphic data (as mean age value of the known geochronologic range of each OTU) were obtained from the primary literature. Where published ages were given in stratigraphic units (e.g. stage or epoch), the dates for the relevant unit were taken from the ICS/IUGS International Stratigraphic Chart [50]. Analyses were conducted using only a single age constraint for the tree, that consisted of the maximum age of the root (Eusauropoda) set at 201 Ma (the Triassic-Jurassic boundary), as this value substantially pre-dates the earliest robust record of eusauropods [51]. Accordingly, root ages of the trees were sampled along a uniform range between 168.8 Ma (the age of the oldest known included OTU, i.e., *Omeisaurus*) and 201 Ma. The monophyly of the ingroup (i.e., the clade including all OTUs with the exclusion of *Shunosaurus*) was enforced, but no internal ingroup topologies were constrained. The BEAST analysis involved 4 replicate runs (with different random starting trees and random number seeds). Each of the replicate runs involved 10 million steps with sampling every 1000 generations, with a burning of 2 million steps. Convergence (stationarity) in numerical parameters was identified using Tracer [52]. The Maximum Clade Credibility Tree (MCCT) resulted from the Bayesian analysis was used as a temporally calibrated phyletic framework for palaeobiogeographic reconstruction, inferring ancestral geographic placement of nodes using RASP (Reconstruct Ancestral State in Phylogenies, [53]). The distribution range of selected sauropod taxa was *a priori* divided into five areas: Asia (A), Europe (B), North America (C), Africa (D), and South America (E). Each terminal taxon was scored for the geographic area character state according to the continent(s) it was recovered in (e.g., *Apatosaurus* was scored as “C”, whereas *Tataouinea* was scored as “D”). Biogeographic inferences on the phylogenetic frameworks were obtained by applying statistical dispersal-vicariance analysis (S-DIVA) and Bayesian Binary Markov Chain Monte Carlo (BBM) analysis [53, 54]. S-DIVA and BBM methods suggest possible ancestral ranges at each node and also calculate probabilities of each ancestral range at nodes. The analyses performed ten Markov Chain Monte Carlo chains of 50000 cycles each, sampling every 100 trees. Chain temperature was set at 0.1. State frequencies were set as estimated and among-site rate variation was set using the gamma parameter. The first 20% of the recovered trees were discarded and the remaining trees were used to infer ancestral range distribution at nodes. In the S-DIVA analyses, direct range dispersal constraints were enforced, excluding those routes considered as not plausible based on Jurassic and Cretaceous palaeogeographic reconstructions ([55–62] and references therein; see S1 File). In both analyses, time-events algorithm [53] was used to infer the total number of dispersal and vicariance events in rebbachisaurid evolution.

## Results

### Parsimony analyses

**Fig 21:**
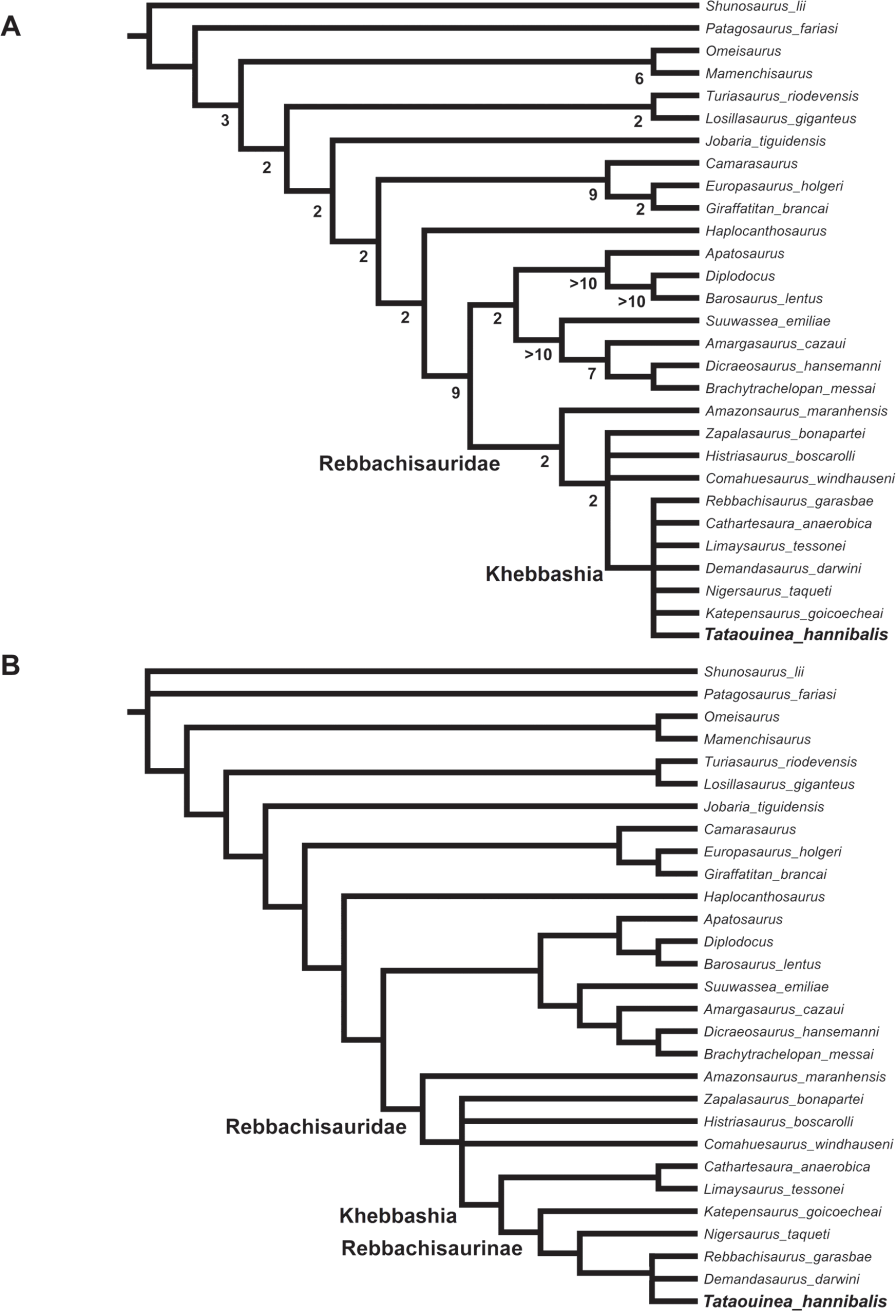
Phylogenetic relationships among rebbachisaurids. Strict consensus topology under equal weighting (A) and under implied weighting (B) of the shortest trees recovered by the parsimony analyses of the dataset. Numbers adjacent to nodes in the equally weighted analysis tree indicate Decay Index values >1.

The analysis recovered 18 shortest trees of 501 steps each (Consistency Index excluding uninformative characters = 0.5108; Retention index = 0.6941). The strict consensus of the shortest trees (Fig 21A) supports the monophyly of Rebbachisauridae, and its placement as sister group of Flagellicaudata (the diplodocid-dicraeosaurid clade), as in all previous analyses of the group. The Brazilian rebbachisaurid *Amazonsaurus* was recovered as the basalmost member of the clade. The relationships among the other rebbachisaurids were poorly resolved: *Comahuesaurus*, *Histriasaurus* and *Zapalasaurus* were recovered in an unresolved polytomy with Khebbashia. Exploration of the alternative shortest topologies indicates that *Histriasaurus*, *Cathartesaura* and *Rebbachisaurus* acted as “wildcard” OTUs, with several alternative placements among a backbone topology formed by the other taxa. When the “wildcard” OTUs are pruned *a posteriori* from the topologies, the other rebbachisaurids form a pectinate series of progressively closer sister taxa to *Tataouinea*: *Amazonasaurus*, *Zapalasaurus*, *Comahuesaurus*, *Katepensaurus*, *Nigersaurus* and *Demandasaurus*. Among the “wildcard” OTUs, it is noteworthy that all alternative placements of *Rebbachisaurus* place it as closer to *Nigersaurus* than *Limaysaurus*, a result supporting the synonymy between Nigersaurinae and Rebbachisaurinae (see Systematic Palaeontology section above).

Nodal support among Rebbachisauridae was relatively low, due to the inclusion of fragmentary OTUs (Decay Index = 2). When the wildcard OTUs were pruned *a posteriori* from calculation, the nodal support of Rebbachisauridae resulted stronger (Decay Index = 6).

We tested whether the low resolution among rebbachisaurids was biased by conflict among differently homoplastic characters, performing implied weighting analyses [63, 64]. The analyses using TNT followed the same protocol of the first analysis, with the *k* parameter (which determines how strongly homoplasious characters are downweighted; see [63] set alternatively as = 3 (default value in TNT) [47], k = 1 (homoplasious characters more strongly downweighted) and k = 9 (homoplasious characters less strongly downweighted). The first analysis (*k* = 3) found three shortest topologies, the strict consensus of which resolving the relationships among Khebbashia (Fig 21B). The Limaysaurinae clade, including *Limaysaurus* and *Cathartesaura*, resulted sister taxon of Rebbachisaurinae (Nigersaurinae of [31]), the latter including *Katepensaurus* as basalmost rebbachisaurine, and *Nigersaurus* as sister taxon of a tricotomy including *Demandasaurus*, *Rebbachisaurus* and *Tataouinea*. Setting *k* = 1 and *k* = 9 produced identical results to the analysis with *k* = 3, indicating that the above discussed relationships are not biased by *a priori* assumptions on character weighting.

### Bayesian analyses

**Fig 22:**
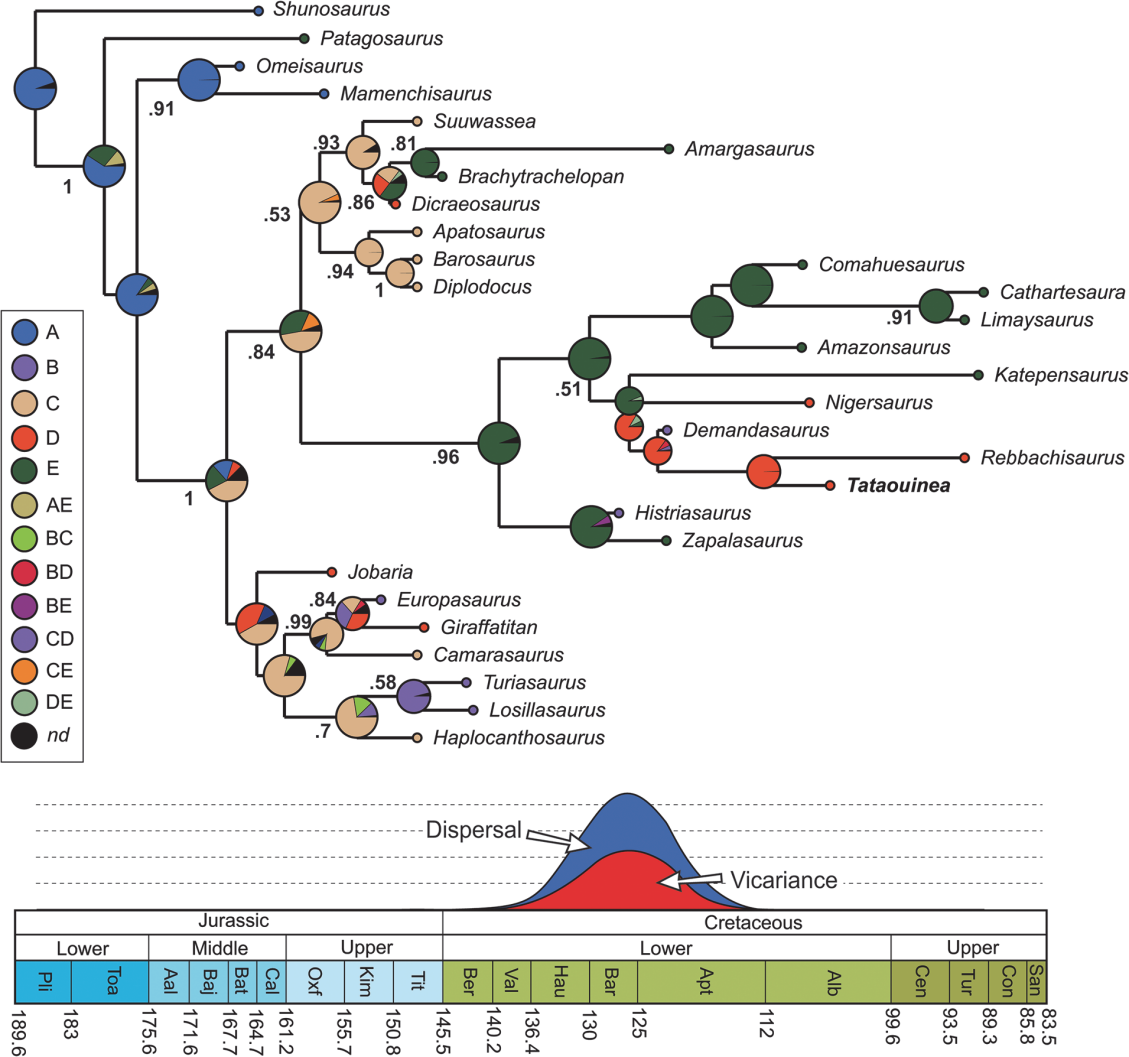
BBM palaeogeographyc analysis of Rebbachisauridae. Time-calibrated palaeobiogeography of eusauropods focusing on rebbachisaurids (above) and result of the time-event algorithm test on Rebbachisauridae (below), based on the BBM analysis of the MCCT recovered by Bayesian inference. Values at nodes indicate posterior probability values >0.5. Abbreviations: A, Asia; B, Europe; C, North America; D, Africa; E, South America. Black circles indicate uncertain optimization.

**Fig 23:**
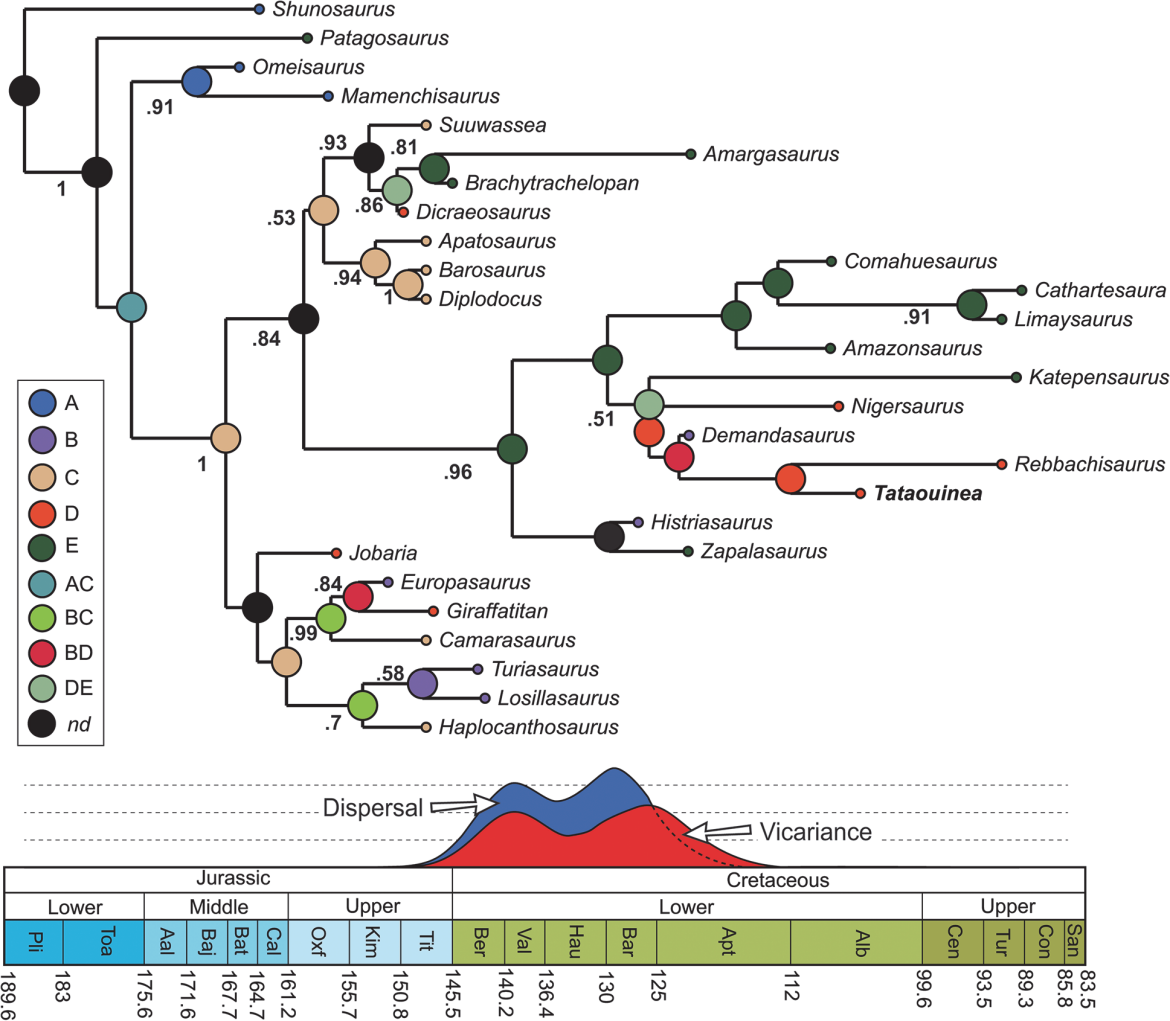
S-DIVA palaeogeographyc analysis of Rebbachisauridae. Time-calibrated palaeobiogeography of eusauropods focusing on rebbachisaurids (above) and result of the time-event algorithm test on Rebbachisauridae (below), based on the S-DIVA analysis of the MCCT recovered by Bayesian inference. Values at nodes indicate posterior probability values >0.5. Abbreviations: A, Asia; B, Europe; C, North America; D, Africa; E, South America. Black circles indicate uncertain optimization.

The MCCT found shows a topology overall comparable to the results of parsimony analyses (topology shown in Figs 22 and 23). Among non-rebbachisaurid OTUs, the most relevant difference from the parsimony-based analysis was the placement of turiasaurians and *Haplocanthosaurus* among macronarian neosauropods instead of, respectively, as a basal eusauropod branch and the basalmost diplodocimorphs. Nevertheless, the basal macronarian nodes including the above-mentioned taxa showed low posterior probability values, and should be considered as tentative. Since an evaluation of non-diplodocoid relationships was beyond the aims of our analysis, and given the small sample among non-diplodocoids, these conflicting interpretations between parsimony and Bayesian analyses are not further discussed here (see [65] for a discussion of turiasaurian placement among Eusauropoda). The monophyly of both rebbachisaurid-flagellicaudatan node and Rebbachisauridae was well supported by the model (posterior probability values, *pp*, of, respectively, 0.84 and 0.96). Although most of the recovered rebbachisaurid nodes show relatively low *pp*, the topology agrees with the results of the parsimony analysis in placing *Histriasaurus* and *Zapalasaurus* as basal rebbachisaurids not members of Khebbashia, in placing *Cathartesaura* as sister taxon of *Limaysaurus*, in recovering Rebbachisaurinae with the same inclusiveness found in the implied weighting parsimony analyses (above), with *Katepensaurus* as basalmost rebbachisaurine, and *Nigersaurus* as sister taxon of the clade including *Demandasaurus*, *Rebbachisaurus* and *Tataouinea*. The Bayesian and parsimony analyses differ in the placements of *Amazonsaurus* and *Comahuesaurus* as basal members of Limaysaurinae in the Bayesian topology. It is noteworthy that the results of a parsimony analysis enforcing *Amazonsaurus* and *Comahuesaurus* as basal limaysaurines (as resulted in the Bayesian analysis) produced shortest topologies only four steps longer than the unforced topologies, a step difference not statistically significant (*p* = 0.12, n = 8) [66], suggesting that these taxa act as “wildcard” OTUs with placement biased by the analytical method followed.

Bayesian analysis simultaneously estimated topology and timing of cladogenetic events. The resulted tree (Figs 22 and 23) placed the macronarian-diplodocimorph divergence at about 170 Ma, the origin of the rebbachisaurid lineage at about 163 Ma, and the Rebbachisaurinae-Limaysaurinae divergence at about 134 Ma (Hauterivian). Among Rebbachisaurinae, the analysis placed the origin of the lineage leading to African and European taxa at about 130 Ma, the divergence of the lineage leading to *Demandasaurus* at about 127 Ma, and the divergence of the lineages leading to *Rebbachisaurus* and *Tataouinea* at about 116.5 Ma (Aptian).

### Palaeobiogeographic analyses

The BBM analysis (Fig 22) supports South America as the most plausible ancestral area of both rebbachisaurid origin and early diversification of Khebbashia (support value = 0.94). The lineage leading to the Croatian basal rebbachisaurid *Histriasaurus* is therefore interpreted as Gondwanan in origin, with the latter genus resulting from a dispersal event occurred between 134 and 131 Ma. Accordingly, a “Gondwanan—European” range for the last common ancestor of *Histriasaurus* and *Zapalasaurus* is poorly supported (support value = 0.07). Furthermore, South America is also interpreted as the exclusive range of limaysaurine evolution and the area of rebbachisaurine origin and earliest diversification (support values, respectively = 0.99 and = 0.91). The most plausible range of the rebbachisaurine subclade including all taxa more derived than *Katepensaurus* is interpreted as African (support value = 0.86), with a single dispersal event to Europe between 130 and 127 Ma clarifying the distribution of *Demandasaurus* (support value = 0.85). Time-events algorithm for rebbachisaurid evolution inferred a single peak for both dispersal and vicariance events, placed at about 127 Ma (Barremian).

Results of the S-DIVA test (Fig 23) mostly agree with the BBM analysis, with all area reconstructions having support value = 1. South America is once again interpreted as the ancestral area of both rebbachisaurid origin and for khebbashian early diversification. The range of Limaysaurine evolution is restricted to South America, whereas the ancestral area of early rebbachisaurine evolution includes both South America and Africa. Most of subsequent rebbachisaurine evolution is placed in Africa, with an “Euro-African” range for the last common ancestor of *Demandasaurus*, *Rebbachisaurus* and *Tataouinea*. Accordingly, the evolution of the most derived rebbachisaurines is interpreted by vicariance between an European lineage (leading to *Demandasaurus*) and an African lineage (including *Rebbachisaurus* and *Tataouinea*). Time-events algorithm for rebbachisaurid evolution inferred two peaks for both dispersal and vicariance events, placed, respectively, at about 140 (Barresian-Valanginian) and 127 Ma (Barremian).

## Discussion

### The evolution of pelvic and caudal pneumatisation in Rebbachisauridae

Newly acquired skeletal elements of the type specimen of *Tataouinea hannibalis* shows that some pneumatic features previously considered exclusive of diplodocids among diplodocoids (e.g., deep fossae in the ventral surface of anterior and middle caudal centra, lateral elliptical fossae in middle caudal centra; e.g., *Diplodocus* MGGC 8723) were present also in some rebbachisaurids. *Tataouinea* includes camerate, semicamellate and camellate pneumatisations in distinct parts of the axial skeleton (e.g., camerate sacral and caudal centra, semicamellate and camellate sacral and caudal neural arches). The variation in pneumatisation pattern is not limited to non-homologue elements (e.g., centrum vs neural arch), but is variable also within single bone elements (e.g., the posterior sacral neural arches 4 and 5 show both camerate and camellate patterns). In the sacral neural arches, the pattern of external pneumatisation follows a cranio-caudal direction, with cranialmost three vertebrae showing a less complex pattern than the posterior two. The extent of pneumatic features in *Tataouinea* holotype is asymmetrical, with the left side of the vertebrae and the left ischium usually bearing a more extensive and elaborate pattern of fossae and foramina than their right counterpart. Asymmetry in the expression of postcranial skeletal pneumatisation has been reported in other sauropods [30, 67]. The presence of pleurocoels in caudal centra 1 to 6 and of distinct lateral pneumatic excavations in caudal centra 14 to 16, both features absent in caudal vertebrae 7 to 13, represents a pneumatic hiatus as those reported in the tails of diplodocids and brachiosaurids among neosauropods [67], in some basal sauropodomorphs [68], and in the sacrum of at least one non-avian theropod (*Tyrannotitan* [69]).

Shallow lateral fossae are present in the proximal caudal centra of *Comahuesaurus* [44], a feature that may be homologue to (and may represent an incipient stage of) the deep pneumatic foramina penetrating the centra of *Tataouinea*. In [70], authors report an isolated caudal vertebra of a rebbachisaurid from the Cenomanian of Morocco, sharing with *Tataouinea* the presence of pleurocoel on centrum. Although no direct evidence supports (neither dismisses) the referral of that vertebra to the sympatric *Rebbachisaurus* [40], the sister-taxon relationship between *Tataouinea* and *Rebbachisaurus* in the Bayesian analysis may support the presence of caudal pleurocoels in *Rebbachisaurus*. Pneumatic foramina are also present in isolated rebbachisaurid caudal vertebrae from the Upper Cretaceous of Argentina that may be referred to *Katepensaurus* [71] (see below), a taxon placed among the basal rebbachisaurines in our phylogenetic analyses.

Fanti et al. [1] proposed a “neural arch first” pattern (*sensu* [72]) for the evolution of tail pneumatisation in rebbachisaurids, based on character optimization of osteological correlates of pneumatisation among Rebbachisauridae, and considered this pattern as a saurischian synapomorphy. This pattern for the caudal vertebrae of both diplodocids and brachiosaurids was dismissed based on the distribution of pneumatic fossae along well preserved caudal series [67]. The complete middle caudal series of *Tataouinea* shows pneumatic fossae on centra and a reduced, albeit still present, pneumatisation on neural arches. Caudal vertebra 16 is the distalmost showing osteological correlates of pneumaticity, present in both centrum (paired fossae on the ventral surface) and neural arch (spinoprezygapophyseal fossa): it is interesting that lateral pneumatisation on both centrum and neural arch is less developed than in the ventral and dorsal surfaces of the vertebra. Although character optimization among Rebbachisauridae suggests a “neural arch first” pattern for the evolution of tail pneumatisation in that clade [1], the distribution of these features in the tail of *Tataouinea* alone does not support a “neural arch” first neither a “centrum first” pattern of pneumatisation.

Iliac internal chambers are reported in *Amazonsaurus* [73] and *Tataouinea*, and may represent a synapomorphy of Rebbachisauridae. An internal pneumatisation of the ischium is present in both *Rebbachisaurus* [40] and *Tataouinea*, but only the latter shows a pneumatic foramen perforating the lateral surface of the iliac peduncle, a feature absent in both *Demandasaurus* and *Rebbachisaurus* [40, 74] and thus autapomorphic for the Tunisian taxon [1].

The body length of *Tataouinea hannibalis* type specimen is estimated in 12 meters, comparable to other rebbachisaurids and small-bodied macronarians [40]. Although osteological correlates of postcranial pneumatisation may be overlooked, in particular in the tail vertebrae (as evidenced by the recent re-analysis of known diplodocid and brachiosaurid specimens [67]), pneumatisation seems more extensive among mid- to small-bodied sauropods than in giant forms, challenging the suggested importance of pneumaticity for lightening the skeletons of sauropods [1, 29].

### *Tempo* and mode of rebbachisaurid evolution

Data presented in this study support a Middle-Late Jurassic origin of the rebbachisaurid lineage as well as the presence of this clade in South America from no later than the Berriasian-Valanginian to the Turonian (e.g., [43]). Rebbachisaurid teeth from Barremian beds of the La Amarga Formation [75] predating the oldest bone evidence of this clade in South America, also support this scenario. By the Jurassic-Cretaceous boundary, all Laurasian diplodocoids (Flagellicaudata) went extinct [76]. Only two flagellicaudatans are known from the earliest Cretaceous (Berriasian-Barremian): the dicraeosaurid *Amargasaurus* [77] and the diplodocine *Leinkupal* [76], both from South America. This geographic pattern may indicate that South American diplodocoids (both rebbachisaurids and flagellicaudatans) were not systematically affected by the Late Jurassic diversity crisis seen in the Northern Hemisphere (see also [78–80]). All known post-Barremian diplodocoids are rebbachisaurids and analyses presented here indicate that both limaysaurine and rebbachisaurine lineages were present in Patagonia until the Cenomanian-Turonian. Isolated rebbachisaurid remains from the Cenomanian-Turonian of central Patagonia including an anteriormost caudal vertebra with pleurocoel on centrum, a distinct hyposphenal ridge and a transverse interprezygapophyseal ridge are also reported [43]. That combination of features is exclusively present in Rebbachisaurinae [42] and supports the referral of that material to the latter clade, eventually to *Katepensaurus* or to a new taxon [43].

The relatively low support for several nodes recovered by the abovementioned analyses is probably biased by the decision to include extremely fragmentary taxa in the ingroup (e.g., *Histriasaurus*). Nevertheless, for this study we consider taxon sample completeness as more significant in the analysis of macroevolutionary and palaeogeographic patterns than the mere nodal support of the chosen phylogenetic framework. Fragmentary taxa, in fact, may provide both temporal (stratigraphic) and spatial (geographic) information unavailable from the arbitrary subset of the best preserved taxa. Although based on a phylogenetically weak topology, the time-calibrated hypothesis presented here represents a testable scenario constraining the *tempo* and mode of rebbachisaurid origin and evolution within a discrete stratigraphic and geographic range.

## Conclusion

In this paper we present a detailed description of the osteology of the type specimen of *Tataouinea hannibalis*, including newly acquired material. Caudal vertebrae 7–17 were collected as fully articulated elements, are exquisitely preserved and provide additional information on rebbachisaurid tail morphology as well as on the development of pneumatization in the caudosacral region within rebbachisaurid sauropods. Caudal and pelvic synapomorphies support the referral of *Tataouinea* to Rebbachisaurinae, here considered as a senior synonym of Nigersaurinae. Time-calibrated phylogeny of Rebbachisauridae indicates a Middle Jurassic origin of the clade, a South American root of Rebbachisaurid radiation, and an expansion to Africa and Europe of Rebbachisaurinae in the earliest part of the Cretaceous.

The time-calibrated phylogeny presented here suggest a rapid cladogenesis of Rebbachisauridae during the Berriasian-Barremian and at least two independent dispersal phases from South America to Africa and Europe approximately between 135 and 130 Ma, one represented by the lineage leading to *Histriasaurus*, the other represented by the rebbachisaurines. Both vicariance and dispersal rates inferred from the known distribution of rebbachisaurids suggest that the Early Cretaceous was the main phase of their expansion, and that this clade rapidly radiated from South America to Africa and then to Europe during a relatively short time interval. *Nigersaurus* represents a pivotal taxon in this scenario, as it is the best known rebbachisaurid, and the basalmost member of the “Euro-African” subclade of Rebbachisaurinae. According to both Bayesian and S-DIVA palaeogeographic analyses, the latter subclade originated from a dispersal event from South America to Africa. Furthermore, both models concur in placing the origin of *Nigersaurus* lineage before the dispersal event to Europe leading to *Demandasaurus* (Bayesian scenario) or the establishment of an “Euro-African” bioprovince including the latter taxon and the “*Rebbachisaurus*-*Tataouinea*” clade (S-DIVA scenario). Both models agree in interpreting the lineage leading to *Tataouinea* as restricted to North Africa. The persistence of both basal forms in South America (i.e., *Katepensaurus*) and derived forms in Africa (i.e., *Rebbachisaurus*) during the early Late Cretaceous suggests that Rebbachisaurinae was the most successful and widely distributed group of rebbachisaurids.

## Supporting Information

S1 File Supporting Material. **Figure A1.** Skeletal reconstruction of *Tataouinea hannibalis*, with missing elements based on other known nigersaurines. **Figure A2.** A. Spars cloud point and camera alignment; B, dense cloud reconstruction based on 64.579.143 points; C. 3D polygonal mesh bas on 12.963.775 faces. D. 3D model texturized reconstruction. **Figure A3.** Photogrammetric 3D surface reconstruction of the 2013 quarry. A, virtual 3D reconstruction of the main quarry elaborated from field pictures. B, detail of the sedimentological structures as reconstructed on the 3D model. Some of the digitized elements, including clinoforms, are less than 1 cm in thickness. **Figure A4.** Morphometric measurement landmarks for caudal vertebrae. f: fragmentary, lost or extremely damaged; h: overall vertebra height; hc: anterior centrum height; hcm: mid-length centrum height; hs: Neural spine height; hsc: neural arch height excluding spine; i: incompletely preserved; l: centrum length; wc: anterior centrum width (measurement taken at centrum mid-height); wm: mid-lenght centrum width (measurement taken at centrum mid-height). **Table A1.** Direct range dispersal routes constrained in S-DIVA analyses. Area abbreviations: A, Asia; B, Europe; C, North America; D, Africa; E, South America.
(DOC)

S1 Fig *Tataouinea hannibalis* 5th caudal vertebra, neural arch. (PDF)

S2 Fig *Tataouinea hannibalis* 5^th^ caudal vertebra, centrum. (PDF)

S3 Fig *Tataouinea hannibalis* 6^th^ caudal vertebra. (PDF)

S4 Fig *Tataouinea hannibalis* 7^th^ caudal vertebra (PDF)

S5 Fig *Tataouinea hannibalis* 8^th^ and 9^th^ caudal vertebrae (PDF)

S6 Fig *Tataouinea hannibalis* 10^th^ caudal vertebra (PDF)

S7 Fig *Tataouinea hannibalis* 11^th^ caudal vertebra (PDF)

S8 Fig *Tataouinea hannibalis* 12^th^ caudal vertebra (PDF)

S9 Fig *Tataouinea hannibalis* 13^th^ and 14^th^ caudal vertebrae (PDF)

S10 Fig *Tataouinea hannibalis* 15^th^ caudal vertebra (PDF)

S11 Fig *Tataouinea hannibalis* 16^th^ caudal vertebra (PDF)

S12 Fig *Tataouinea hannibalis* 17^th^ caudal vertebra (PDF)

S13 Fig *Tataouinea hannibalis* left ischium (PDF)

S14 Fig *Tataouinea hannibalis* right ischium (PDF)
