# Supplementary material for: New Information on Tataouinea hannibalis from the Early Cretaceous of Tunisia and Implications for the Tempo and Mode of Rebbachisaurid Sauropod Evolution
Source: PLoS One. 2015 Apr 29;10(4):e0123475. doi: 10.1371/journal.pone.0123475 (PMC4414570; doi:10.1371/journal.pone.0123475)
Supplement: S1 File — Figure A1. Skeletal reconstruction of Tataouinea hannibalis, with missing elements based on other known nigersaurines. Figure A2. A. Spars cloud point and camera alignment; B, dense cloud reconstruction based on 64.579.143 points; C. 3D polygonal mesh bas on 12.963.775 faces. D. 3D model texturized reconstruction. Figure A3. Photogrammetric 3D surface reconstruction of the 2013 quarry. A, virtual 3D reconstruction of the main quarry elaborated from field pictures. B, detail of the sedimentological structures as reconstructed on the 3D model. Some of the digitized elements, including clinoforms, are less than 1 cm in thickness. Figure A4. Morphometric measurement landmarks for caudal vertebrae. f: fragmentary, lost or extremely damaged; h: overall vertebra height; hc: anterior centrum height; hcm: mid-length centrum height; hs: Neural spine height; hsc: neural arch height excluding spine; i: incompletely preserved; l: centrum length; wc: anterior centrum width (measurement taken at centrum mid-height); wm: mid-lenght centrum width (measurement taken at centrum mid-height). Table A1. Direct range dispersal routes constrained in S-DIVA analyses. Area abbreviations: A, Asia; B, Europe; C, North America; D, Africa; E, South America. (DOC) [file pone.0123475.s001.doc]

**SUPPLEMENTARY MATERIAL OF**

**New Information On *Tataouinea hannibalis* From The Early Cretaceous of Tunisia And Implications For The *Tempo* And Mode Of Rebbachisaurid Sauropod Evolution**

Federico Fanti ^a,b*^, Andrea Cau ^a,b^, Luigi Cantelli ^a^, Mohsen Hassine ^c^, Marco Auditore^d^

^a^ Dipartimento di Scienze Biologiche, Geologiche e Ambientali, Alma Mater Studiorum, Università di Bologna, Bologna, Italy

^b^ Museo Geologico Giovanni Capellini, Alma Mater Studiorum, Università di Bologna, Bologna, Italy

^c^ Office National Des Mines, Service Patrimoine Géologique, Tunis, Tunisia.

^d^ Museo Paleontologico Cittadino, Monfalcone, Gorizia, Italy


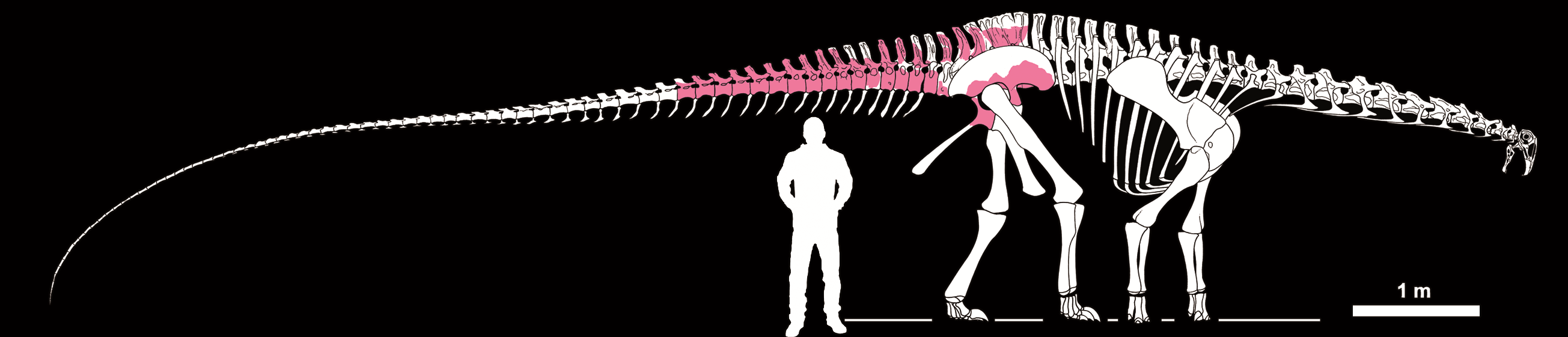


**Figure A1.** Skeletal reconstruction of *Tataouinea hannibalis*, with missing elements

based on other known nigersaurines.

**Photogrammetric reconstruction of the 2013 quarry of *Tataouinea hannibalis***

A detailed quarry map of ongoing excavations is commonly a critical factor as field conditions and timing can vary unexpectedly. During the field activity in 2013 we performed a single photographic survey of the main excavation area taking approximately 60 high resolution photographs of which 43 were consequently selected for a photogrammetric reconstruction. In particular, we included two sets of images (Nikon V2 camera, fixed focal length): a first set realized with a lens of 18.5mm focal length (35mm equivalent: 50 mm) and a second with focal length of 10 mm (35 mm equivalent to 27 mm). Images were processed using Agisoft Photoscan Professional® combining multi-views 3D reconstruction on steps in sequences. The first stage (camera alignment) matches common points on photographs producing a sparse point cloud and a set of reference camera positions. subsequently, the software reconstructs a 3D polygonal surface (building mesh) from the high density point cloud. Once the final mesh is reconstructed, it can be fully textured and also used to generate high-resolution orthophotographs.

**
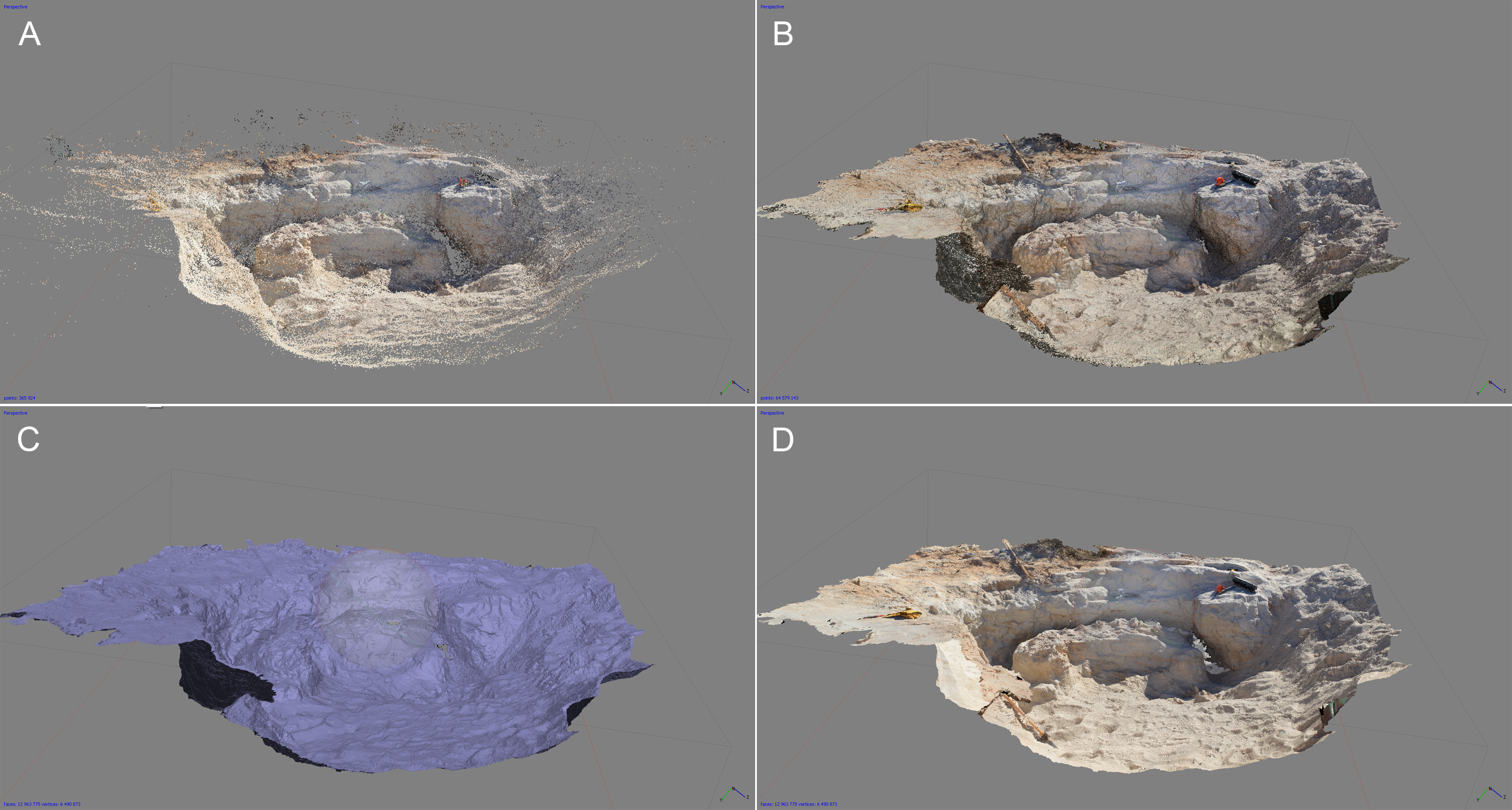
**

**Figure A2.** 3D photogrammetric reconstruction of the *T. hannibalis* main quarry. A. Spars cloud point and camera alignment; B, dense cloud reconstruction based on 64.579.143 points; C. 3D polygonal mesh bas on 12.963.775 faces. D. 3D model texturized reconstruction.


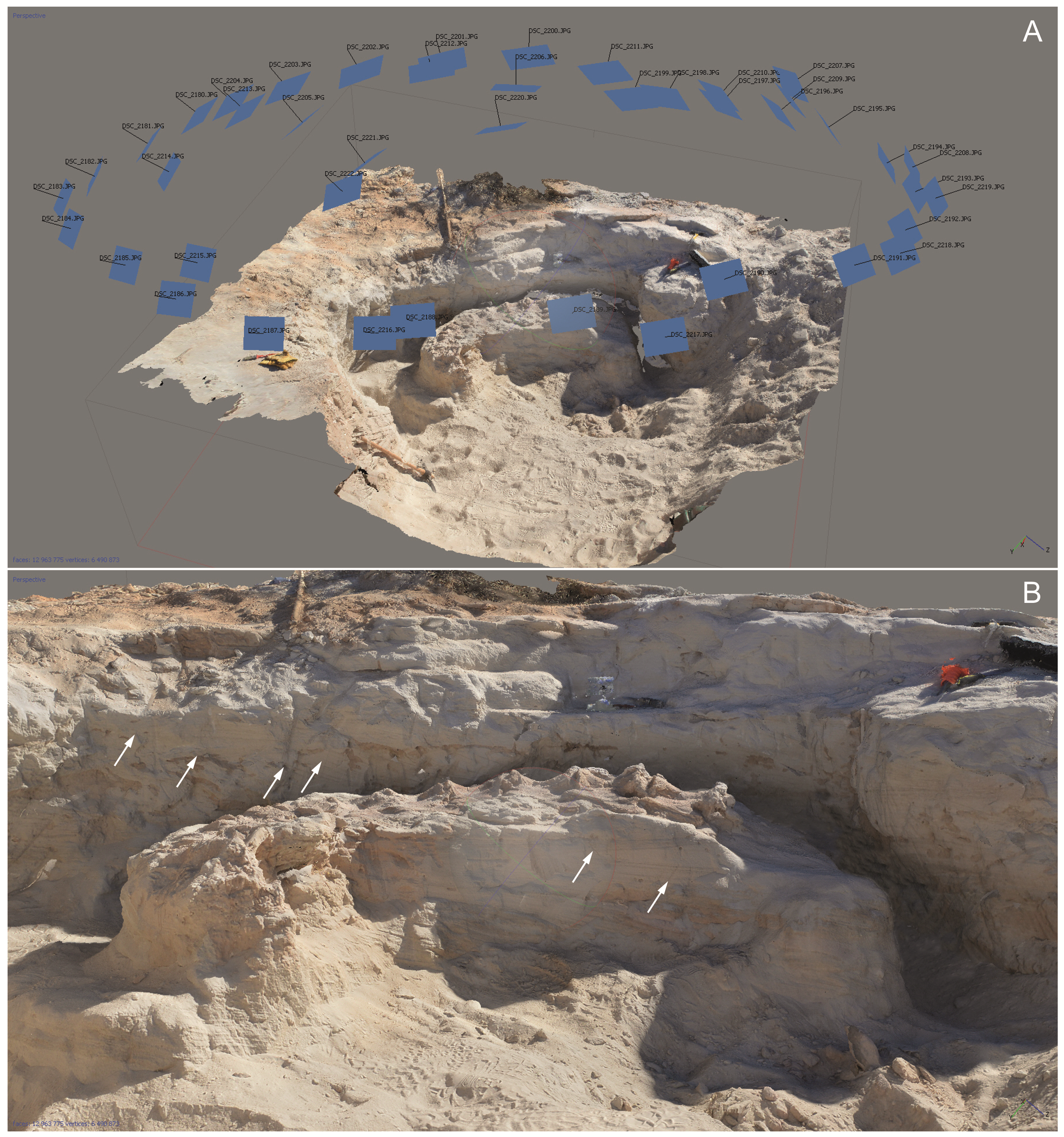


**Figure A3.** Photogrammetric 3D surface reconstruction of the 2013 *T. hannibalis* main quarry. A, virtual 3D reconstruction of the main quarry elaborated from field pictures. B, detail of the sedimentological structures as reconstructed on the 3D model. Some of the digitized elements, including clinoforms, are less than 1 cm in thickness.

**Measurements (cm) of caudal vertebrae 6-17 of *Tataouinea hannibalis* holotype.**

| **Specimen** | **position** | **l** | **wc** | **wm** | **hc** | **hcm** | **h** | **hs** | **hsc** |
| --- | --- | --- | --- | --- | --- | --- | --- | --- | --- |
| **ONM DT37** | 6th | 14 | 12 | 3 | 12 | 14 | 30 | f | 15 |
| **ONM DT38** | 7th | 13 | 13 | f | 18 | 14 | 30 | f | 13i |
| **ONM DT39** | 8th | 13 | 10 | 3 | 14.5 | 11.5 | 37 | f | 22i |
| **ONM DT40** | 9th | 19 | 10 | 4 | 14,5 | 14 | 37 | f | 23i |
| **ONM DT41** | 10th | 16 | 10.5 | 4 | 16 | 13 | 38 | 6 | 22 |
| **ONM DT42** | 11th | 23 | 12 | 5 | 14.5 | 13 | 32 | 6.5 | 16 |
| **ONM DT43** | 12th | 23 | 11 | 4,5 | 13 | 12 | 31 | 6.5 | 16 |
| **ONM DT44** | 13th | 24 | 11 | 4 | 14 | 10 | 30 | 6.5 | 15.5 |
| **ONM DT45** | 14th | 21 | 11 | 3.5 | 13 | 11 | 26.5 | 6 | 13.5 |
| **ONM DT46** | 15th | 24 | 13 | 5,5 | 13 | 9 | 27 | 7 | 13 |
| **ONM DT47** | 16th | 25 | 14 | 6 | 14 | 10 | 26 | 5 | 13 |
| **ONM DT48** | 17th | 22 | 12 | 6 | 11 | 9 | 17 | 5 | 6,5 |


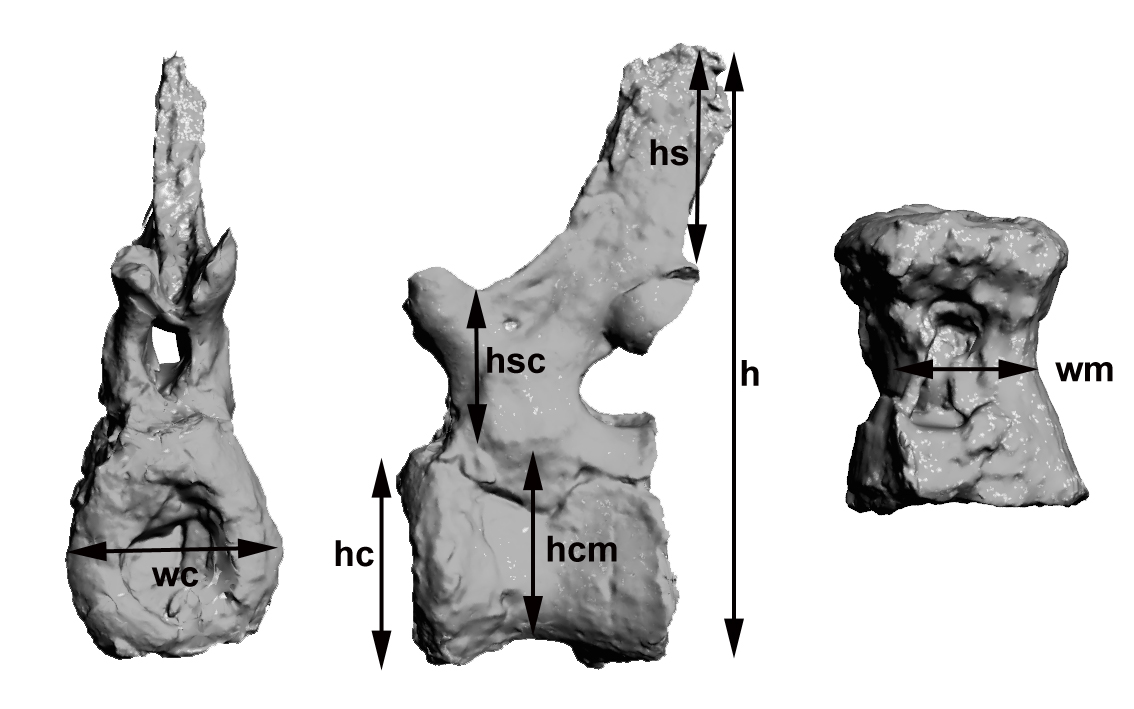


**Figure A4. Morphometric measurement landmarks for caudal vertebrae.**

**f**: fragmentary, lost or extremely damaged; **h**: overall vertebra height; **hc**: anterior centrum height; **hcm**: mid-length centrum height; **hs**: Neural spine height; **hsc**: neural arch height excluding spine; **i**: incompletely preserved; **l**: centrum length; **wc**: anterior centrum width (measurement taken at centrum mid-height); **wm**: mid-lenght centrum width (measurement taken at centrum mid-height).

| **From/to** | **A** | **B** | **C** | **D** | **E** |
| --- | --- | --- | --- | --- | --- |
| **A** | allowed | allowed | allowed | not allowed | not allowed |
| **B** | allowed | allowed | allowed | allowed | not allowed |
| **C** | allowed | allowed | allowed | not allowed | not allowed |
| **D** | not allowed | allowed | not allowed | allowed | allowed |
| **E** | not allowed | not allowed | not allowed | allowed | allowed |

**Table A1.** Direct range dispersal routes constrained in S-DIVA analyses. Area abbreviations: A, Asia; B, Europe; C, North America; D, Africa; E, South America.

**Phylogenetic data**

**New characters added to the data set of [44]**

(342) Middle and posterior dorsal vertebrae, parapophysis dorsoventral position relative to prezygapophysis: at the same level (0); more dorsally (1). [40].

(343) Anterior caudal vertebrae, ventral interprezygapophyseal lamina: absent (0); present (1). [41].

(344) Anterior caudal vertebrae, lamina bisecting the prezygapophyseal centrodiapophyseal fossa: absent (0); present (1). [43].

(345) Mid- and posterior dorsal neural arches, large pneumatic foramina that passes all the way through the neural arch anteroposteriorly: absent (0), present (1). [40].

(346) Posterior dorsal neural arches, centroprezygapophyseal lamina (cprl), shape: single (0); divided (1). [40].

**Age of included OTUs (in Mya)**

*Shunosaurus lii* 166.9 [78]

*Patagosaurus fariasi* 162.3 [78]

*Omeisaurus* 168.8 [78]

*Mamenchisaurus* 160.4 [78]

*Turiasaurus riodevensis* 146.2 [79]

*Losillasaurus giganteus* 145.5 [78]

*Jobaria tiguidensis* 159.6 [78]

*Haplocanthosaurus* 151.1 [78]

*Camarasaurus* 151.1 [78]

*Europasaurus holgeri* 154.7 [80]

*Giraffatitan brancai* 150.4 [78]

*Amazonsaurus maranhensis* 112.8 [31]

*Zapalasaurus bonapartei* 126.3 [31]

*Histriasaurus boscarolli* 131.0 [78]

*Comahuesaurus windhauseni* 112.7 [44]

*Rebbachisaurus garasbae* 96.6 [78]

*Cathartesaura anaerobica* 94.7 [31]

*Limaysaurus tessonei* 96.6 [78]

*Demandasaurus darwini* 126.2 [42]

*Nigersaurus taqueti* 112.0 [78]

*Suuwassea emiliae* 151.1 [31]

*Amargasaurus cazaui* 126.0 [78]

*Dicraeosaurus hansemanni* 153.2 [78]

*Brachytrachelopan mesai* 148.6 [31]

*Apatosaurus* 151.1 [78]

*Diplodocus* 151.1 [78]

*Barosaurus lentus* 151.1 [78]

*Katepensaurus goicoecheai* 95.2 [43]

*Tataouinea hannibalis* 110.0 [this study]

**Data matrix**

Shunosaurus_lii

0100100000110110000010000000??000001001??0000001101100000100011000000000??000001100?0000?101?110??011011?201201001000100000010000100?101000110?1?1?0000000000011101???11??000010000010?0001010000000000100100?00?0002001?00???111010100000010??00001000010011011010010101000100000110101100000100000???0?011100110001101?100?10?00111101110111?11111000???

Patagosaurus_fariasi

1???1?00??11???????????????????0???????????????????????????????1?????????????????0???????????1???1011011?000??100100?000000010110100??00000110?001?00000011000110010?011??000010??002??0?0?????000000??1??1????0?0002002?0??????00??11000001?000?00100??????101001001110100????????????11000?01000000010?011100010000001???????????101011?????????????????

Omeisaurus

11001?0000110111000010???000???001010011000001011011?000?1???11100?0?0??00?0?00110000000?1???111??011011010040111230000?000101100100?21000?111001100000100100?11110100111?000210010?2110100?10000000000100100?00?0002001?00???1100001100000??000?0000000100110110100101010001000000111011000001000000010?1111000000001011??001010011111111111111111100??00

Mamenchisaurus

11101?0?00111011000110??0?00???00?010010000?010?1?111000?0???101?00??????0?0???1100???00110111100?011011?1004011123?00020001011001010210012111001101100100?000110021001?1101021001002??0?00?11003000000100100?00?0002002?0????1100?0110?0?0?1????00?0?0????11011010011101001101?0001???1100000??00000010?011100010001101?11001110?11?101?1?1111??1???0????

Turiasaurus_riodevensis

????????????????????????????????????????????????????????????????????????????????????????????????????10110001??11120100000100101?0101??11?1011100010??00000100011101100111100?1100100???????0???02000?0?1????????????????200?????????1?????????????????00??0?10110100????10011110000111??????????????????????????????00??11?101010?11???11???1?10?111??????

Losillasaurus_giganteus

???????????????????????????????????????????????????????????????????10?????0????1??????????????1??1?1101000?0??1012010000000010110100??11110111001101000100?000111011001111100110???????????0???0200?10?100??00000??????1????????????????????????????????????1011010?????1??????????????01?0??010?0?00?11?0????????????????????????????????????????????????

Jobaria_tiguidensis

11001?000011111?010010??0000??10010100?100000101101110???10??11?000??00?00?0?0?1100000???????1100?01101100001?1??221?000000010010100?201010110?0?1?11000001000111011001111000000010021101?10100000??000100?00?100000200100????1?00101101010????01000000????110110100111010001110000111111000?01000000011?0111000100?110111011111001111011111????????????00

Haplocanthosaurus

??????????????????????????????????????????????????????????????????????????????????????????????????????????????101221?001000010010100?01111011100110110001010001100110011101001101100211011?010000000000100100?10000120010???????10??11000211?000100?00?000????????????????????????????111000?01000000011001110001001????????1????????????????????????00?00

Camarasaurus

111010000011111101000000000000100101000101000101101110001101?111000000000010000110000011111111110?011011021010101232?0?10100101101011210011110?001011000001000110121001111000210010?2110111010000000001100100?10000020010000???0?0101101000101001001000000011011010011101001111110011111100000110000101100111000100111011101011100111101111111111111000?00

Europasaurus_holgeri

11101?0?001111?0010010000?????10?1?00?01001?01011?1110000101????100000001110?0?1100???1111?1111101011011?210?01012021001101110110100??100111111011021000001000110011001110100210010021?001?0?0000000000100000010000020010000??0?10??11000001010010010000????101101001110100??11??????1111101?0110000?0110?111000100?1101110101110?11?1???1??1?1??11???????

Giraffatitan_brancai

121010000011111101001000100000100101000100000101101110000101?111000000001110?00110000011111121210?0010110201?0111232?002000111110100?210011111001101020010101011?12100111001021?0111211011?010001000000100100?1000102001000?????10??1101000?00001001100000021010010111101001111111110111110110110000101100112100100111011111?1110011110111?11?1??11?000?00

Amazonsaurus_maranhensis

??????????????????????????????????????????????????????????????????????????????????????????????????????????????????????????????????????1???111????10??0???????????0?1?0111??1???????????????0???000??0?1?0?0?1?1100002?1?00??????10???????????????????????????????????????????????????????0????????????????????????????????????????????????????????????1???

Zapalasaurus_bonapartei

??????????????????????????????????????????????????????????????????????????????????????????????????????????????11?221001?00?????????????????????????????????????????????????????????????????????1001010110?0110010000201100??????????????????????????????????????????????????????????????????????000?0?11?0????????????????????????????????????????????0???

Histriasaurus_boscarolli

??????????????????????????????????????????????????????????????????????????????????????????????????????????????????????????????????????1101??????????10?011??00???00101111?01?010??????????????????????????????????????????????????????????????????????????????????????????????????????????????????????????????????????????????????????????????????????????

Comahuesaurus_windhauseni

????????????????????????????????????????????????????????????????????????????????????????????????????1221?203??????????????????????????11011110?0210220?1?11??0???00111111??1?01??????????0?0?201400000?10?0????100002011?0??????10??1????????????00?001?????10110111?????????????????????????01010000011?0211000100???????????????????????????????????0000

Rebbachisaurus_garasbae

??????????????????????????????????????????????????????????????????????????????????????????????????????210?????????????????????????????1101?110?02?0??01211110011100101111?111010???????1????????????0?1?????111?????????????????????11021?1001001???????????100?????????????????????????????????101?1????1???????????????????????????????????????????1??11

Cathartesaura_anaerobica

??????????????????????????????????????????????????????????????????????????????????????????????????????????????1102210011??0???000100???????????????????????????????????????????????????????????1????0111000?11?100012?1??0???????????1?21110011?1?????????????????????????????????????????????????????????2110001?????????????????????????????????????00??

Limaysaurus_tessonei

???????????1?01????1?1??0000??11??0011111?001?0?10111011?20?????100101010110?101????????????????????12211203??1102210011000010000100??11011110?0210110121011001100010110??1110101?0????????01?014000001100001111000020110011?1??10??11021110011010010011???1101001011110100????????????11???00101100001101211000?001?10??1?11???0??10001111???????????00??

Demandasaurus_darwini

?0?0????????????????????????????????????????????????????????????????????????????10120????????2?2?1?012211203??1102310011000010200100??11??1?10?02????0?1?1110111100101?11??1?01011???????????20140001012101?1010100??0??00??????10??????????????????????????????????????????????????????????????111????1?1211010100??????????????????????????????????11111

Nigersaurus_taqueti

00201?10101100?1??1111??0000??11??0011110??11????0111011?2??????100100010??0?1?1101200100????2301?00122112032?1102210011000010200100?211?11110?02101201111110111100101111?1100101?00???1???????140??1?1?1?0?1111?00120110011?11010??110212100100100?00??????10000101???????????????????110??0???111??????1211010100?????????1????????????????????????11?00

Suuwassea_emiliae

0121???1???2?????????1??01???????00?00??11100?0??1??0?10????????0????0?0?????????1?11????????2?2???1??210????0111221000100101010???1??110?11?????1021?????????????????1?????????1100???????0????1??????????????????????????????????????0000?000????????????????2??????????????????????????????????????????????????????????????????????????????????????????

Amargasaurus_cazaui

???????????2?011?????????100??10?01100011110011111??????????????0011101001?0?111????????????????????????????11110211000??1?1101001?10?01020110?1?1?1100210110?10?00100111?0000101??02??1?????0?0?0??0??????010???0?020??????????????1??0??????0?1??????????1101101001010?00????????????11001??????????????1110?0?001????????1????????????????????????0??00

Dicraeosaurus_hansemanni

0021???1???2??01?????????100???0?011000111100111111?????1?0???10001110100010?11111011????????2311?0011210203111102110000?1?1101101?11201020110?1?1?1100010110110000100111?100010110021?1?0?01100200001110010101000002012?0?1?11?00??1100000???00100??0?????1101101001110??????????????11100001100000??001011100010011101110111110??111111?11????11???01?00

Brachytrachelopan_mesai

??????????????????????????????????????????????????????????????????????????????????????????????????????????????1?0?11???0?????????????201020110?1?1?1?00010110110000100111?1000101????????????????????????????????????????????????????????????????????????????????????????????????????????????????????????????????????????????????????????????????????0???0

Apatosaurus

00201?11111201111?111100101100?0010100011101010110110010020?01?00001000000000101?1?001???????2311?00012102?331111231?00201?1102101?1131000?110?02101100001100111100100111?010010110021?111201100200001111110111000002012?0111111001011000001?0011001?00000?110120100101010011110000111111000011000001?0010111100100111011101111101111111111?1111?11100??00

Diplodocus

00201011111201111111110010110010010100011101010110110010020101100001000010100101110001101101?2311?100121020331111231?00201?0112111?1131000111??02101100001100111100100111?0101101100211111201100211101111111111210003012?0111111001011000000?1001001?0000001101101001110100???????????111000011000000?001011100010111101?1011111001111111111111?1111001?00

Barosaurus_lentus

????????????????????????????????????????????????????????????????????????????????????????????????????????????3?111231?00?00?1112011?11310001110?02101100001100111100100111?01001011002????0?01100211101111111111210003012?011?11?00?01???000????????1??0??????0?00?0????????????????????11??0?11?0???????10111000???11????????1??????????????????????????00

Katepensaurus_goicoecheai

??????????????????????????????????????????????????????????????????????????????????????????????????????????????10022??01????1100???????11???1?1???111??12?1?10???1??1?1?110111?1?1??????????????1????1111000?101??????????????????????????????????????????????????????????????????????????????????????????????????????????????????????????????????????011?0

Tataouinea_hannibalis

????????????????????????????????????????????????????????????????????????????????????????????????????????????????????????????????????????????????????????????????????????????????????211?11?12??121??11121?10111010002?01??????????????????????????????????????????????????????????????11?01?????11101????1????????????????????????????????????????????0???
