# Supplementary figures and images for: New Information on Tataouinea hannibalis from the Early Cretaceous of Tunisia and Implications for the Tempo and Mode of Rebbachisaurid Sauropod Evolution
Source: PLoS One. 2015 Apr 29;10(4):e0123475. doi: 10.1371/journal.pone.0123475 (PMC4414570; doi:10.1371/journal.pone.0123475)

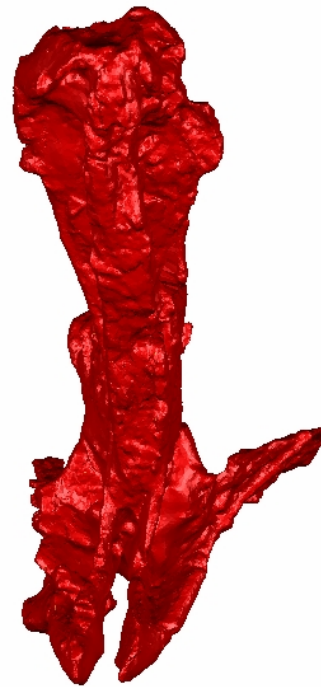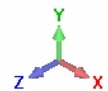

Click on the image to activate the 3D Model.

Supplement: S1 Fig — (PDF) [file pone.0123475.s002.pdf]

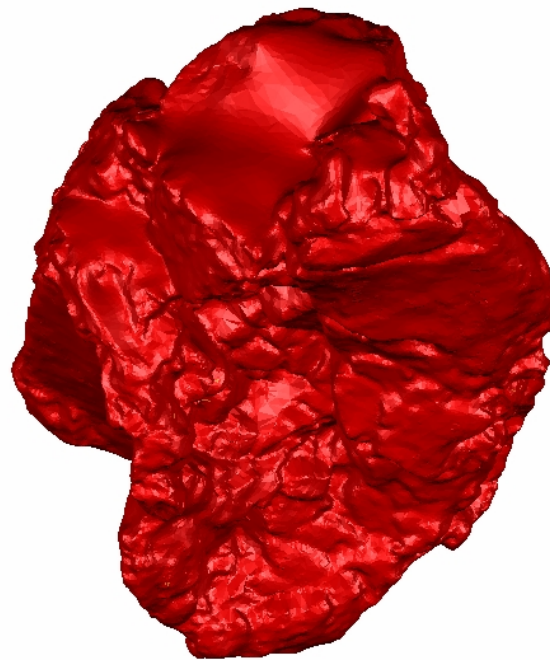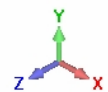

Click on the image to activate the 3D Model.

Supplement: S2 Fig — (PDF) [file pone.0123475.s003.pdf]

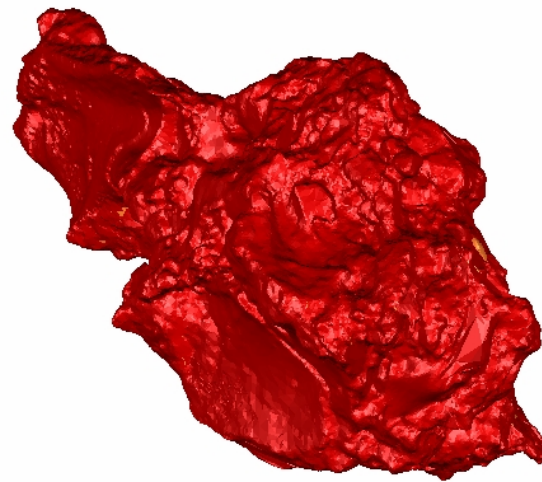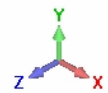

Click on the image to activate the 3D Model.

Supplement: S3 Fig — (PDF) [file pone.0123475.s004.pdf]

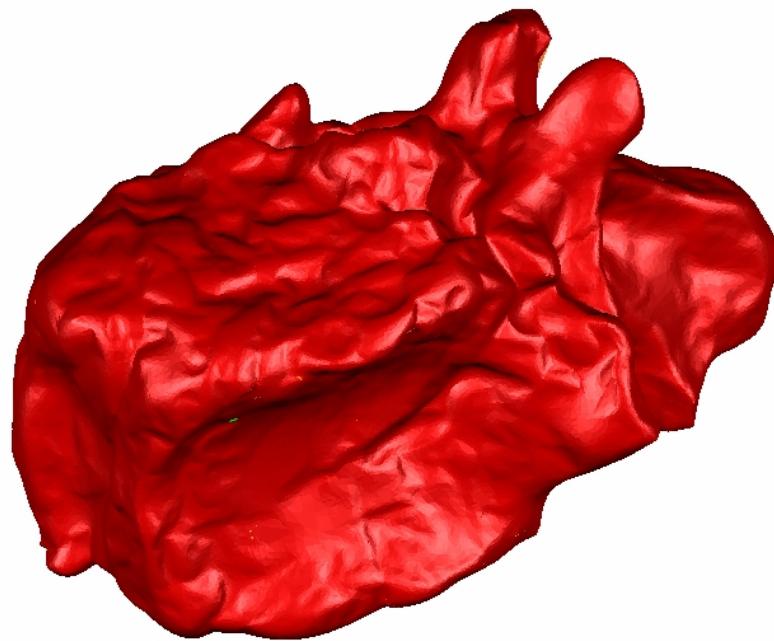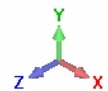

Click on the image to activate the 3D Model.

Supplement: S4 Fig — (PDF) [file pone.0123475.s005.pdf]

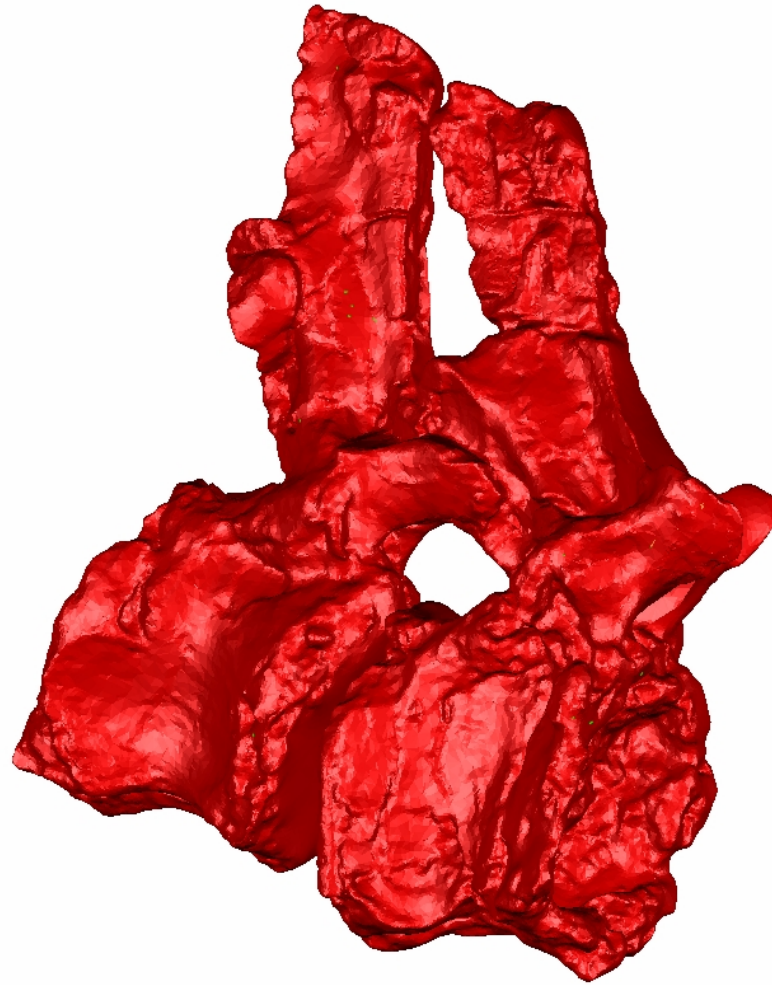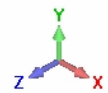

Click on the image to activate the 3D Model.

Supplement: S5 Fig — (PDF) [file pone.0123475.s006.pdf]

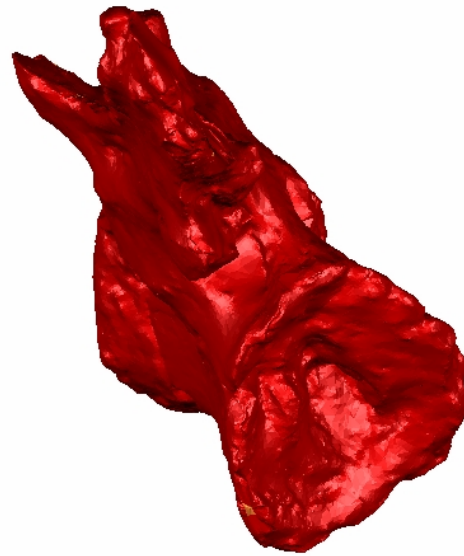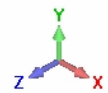

Click on the image to activate the 3D Model.

Supplement: S6 Fig — (PDF) [file pone.0123475.s007.pdf]

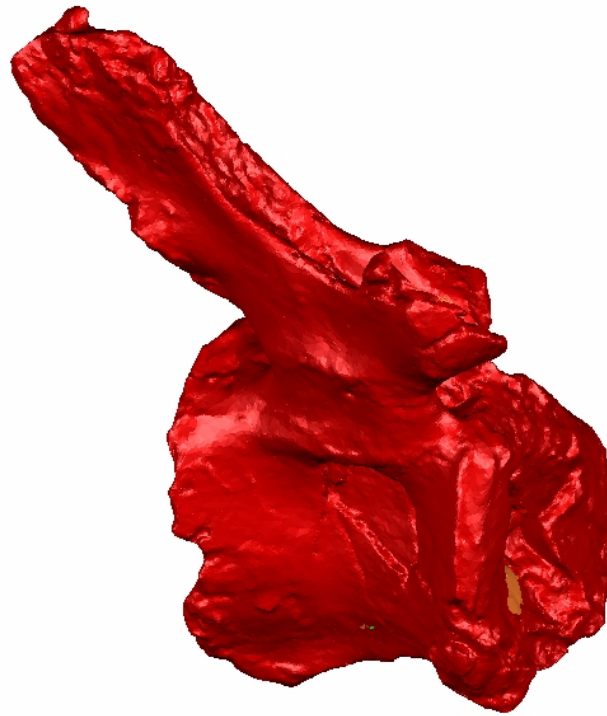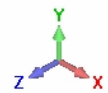

Click on the image to activate the 3D Model.

Supplement: S7 Fig — (PDF) [file pone.0123475.s008.pdf]

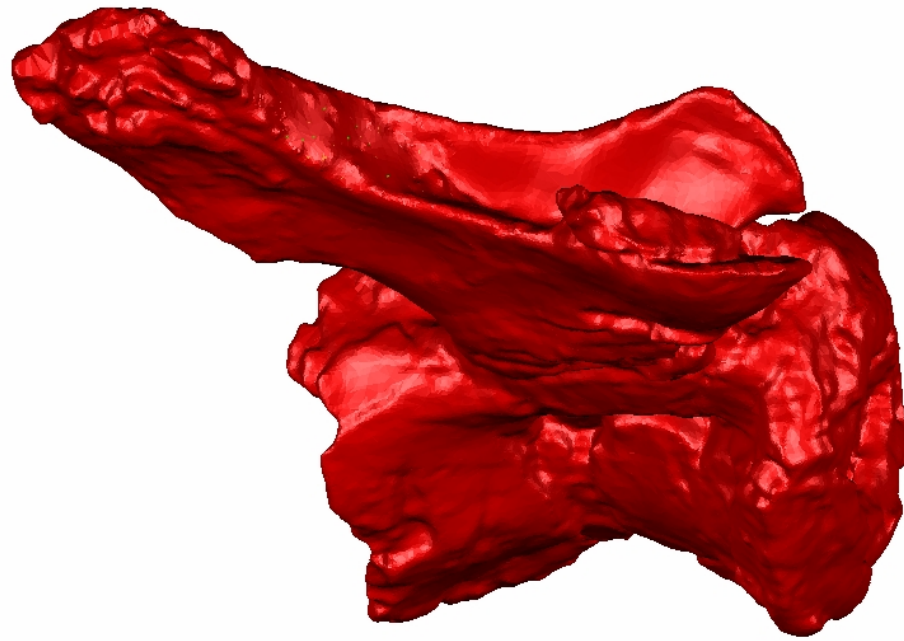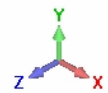

Click on the image to activate the 3D Model.

Supplement: S8 Fig — (PDF) [file pone.0123475.s009.pdf]

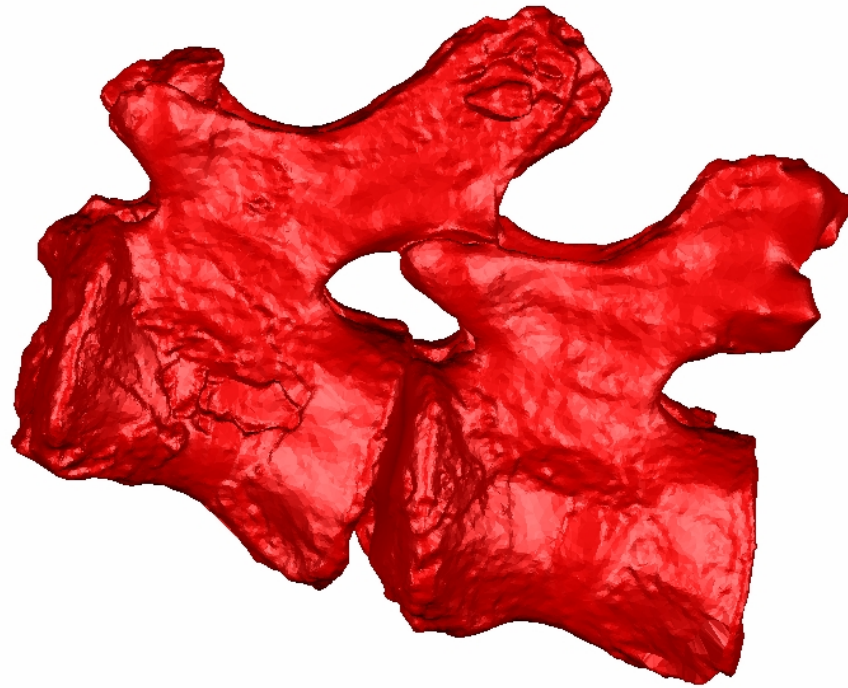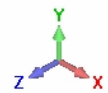

Click on the image to activate the 3D Model.

Supplement: S9 Fig — (PDF) [file pone.0123475.s010.pdf]

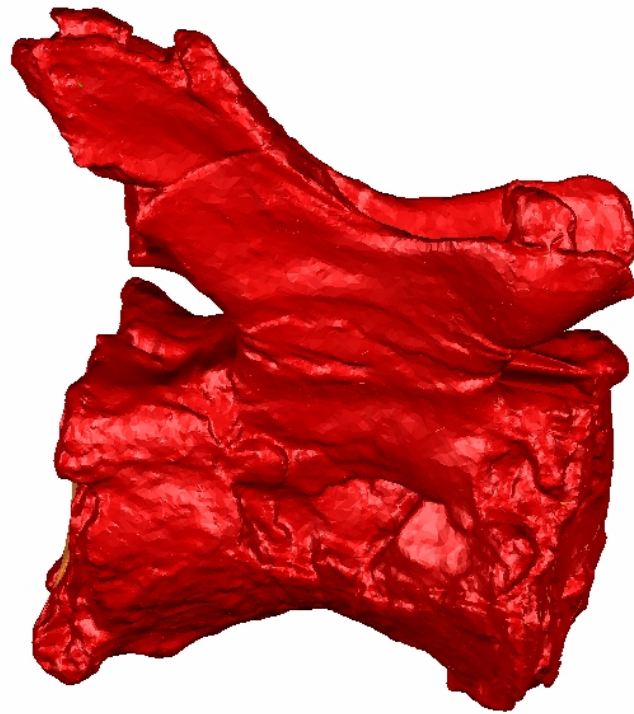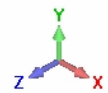

Click on the image to activate the 3D Model.

Supplement: S10 Fig — (PDF) [file pone.0123475.s011.pdf]

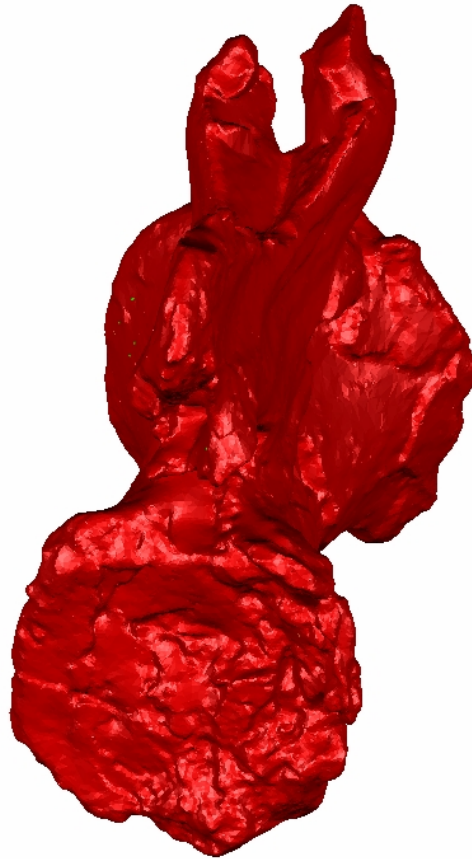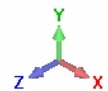

Click on the image to activate the 3D Model.

Supplement: S11 Fig — (PDF) [file pone.0123475.s012.pdf]

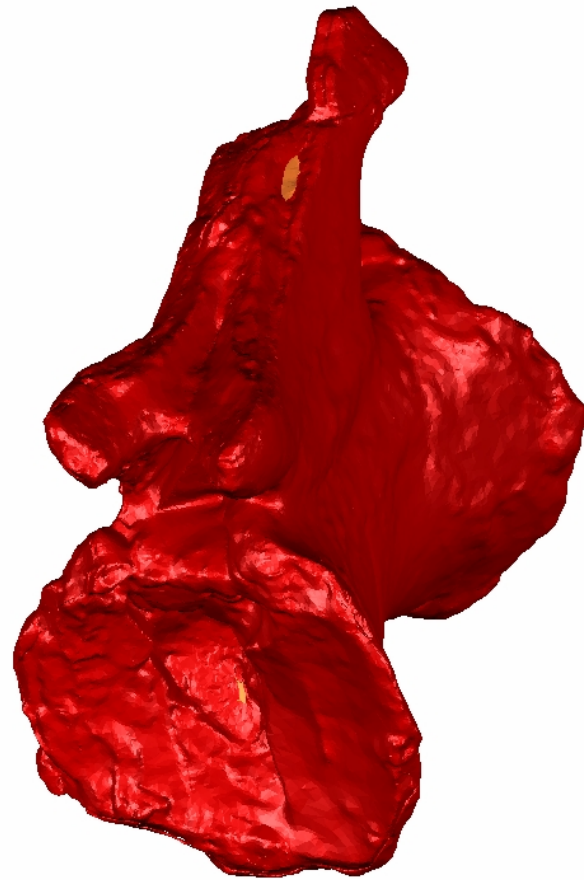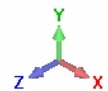

Click on the image to activate the 3D Model.

Supplement: S12 Fig — (PDF) [file pone.0123475.s013.pdf]

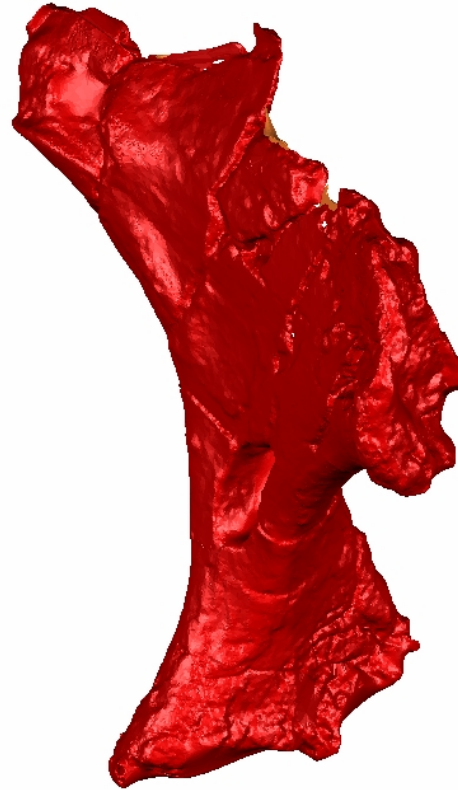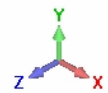

Click on the image to activate the 3D Model.

Supplement: S13 Fig — (PDF) [file pone.0123475.s014.pdf]

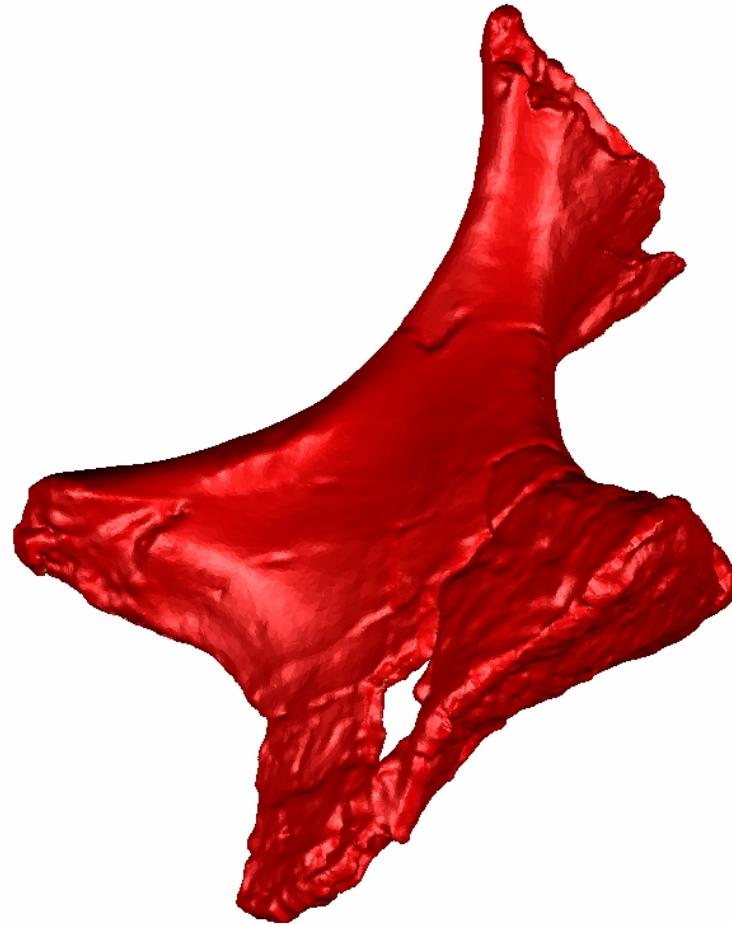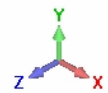

Click on the image to activate the 3D Model.

Supplement: S14 Fig — (PDF) [file pone.0123475.s015.pdf]
